# On the Mechanism of Action of the Antitumor Drug cis-Platin (cis-DDP) and its Second Generation Derivatives

**DOI:** 10.1155/MBD.1995.153

**Published:** 1995

**Authors:** Vasilis Aletras, Denis Hadjiliadis, Nick Hadjiliadis

**Affiliations:** 1 University of Ioannina Department of Chemistry Laboratory of Inorganic and General Chemistry Ioannina 45-110 Greece; 2 University of Toronto Faculty of Medicine 1 King’s College Circle Toronto Ontario H4A lW3 Canada

## Abstract

The present article attempts to summarise the elements of the mechanism of action of the antitumor drug cis¯-Platin presented the last few years. Highlights on the discovery, of the drug and the development of it’s second generation derivatives are presented, as well as the ways that cis¯-DDP reacts with biomolecules as DNA and proteins and their models e.g. nucleosides, nucleotides. Also the hydrolysis data are presented for cis¯-DDP and its’ inactive congener trans¯-DDP, as well as for the second generation drug carboplatin. Finally, usefull conclusions are given from this work, pointing out the unanswered questions about the action of cis¯-DDP as well as its differences in action, in comparison with trans¯-DDP.


### ON THE MECHANISM OF ACTION OF THE ANTITUMOR DRUG cis-PLATIN (cis-DDP) AND ITS SECOND GENERATION DERIVATIVES

Vasilis Aletras[1], Denis Hadjiliadis[2] and Nick Hadjiliadis[1*]

[1] University of Ioannina, Department of Chemistry, Laboratory of Inorganic and General Chemistry, Ioannina, 45-110, Greece
[2] University of Toronto, Faculty of Medicine, 1 King's College Circle, Toronto, Ontario H4A 1W3, Canada

Abstract: The present article attempts to summarise the elements of the mechanism of action of the antitumor drug cis-Platin presented the last few years. Highlights on the discovery of the drug and the development of it's second generation derivatives are presented, as well as the ways that cis-DDP reacts with biomolecules as DNA and proteins and their models e.g. nucleosides, nucleotides. Also the hydrolysis data are presented for cis-DDP and its' inactive congener trans-DDP, as well as for the second generation drug carboplatin. Finally, usefull conclusions are given from this work, pointing out the unanswered questions about the action of cis-DDP as well as its differences in action, in comparison with trans-DDP.

### TABLE OF CONTENTS

1. HISTORICAL BACKROUND
   a. Introduction.
   b. Structure - Activity Relationships.
   c. Binding sites of cis-DDP responsible for cytotoxicity and side toxicities.
   d. Hydrolysis of cis and trans-DDP in aqueous solutions.

2. BINDING MODES OF METAL IONS WITH DNA
   a. Introduction (Description of DNA structures)
   b. Formation of a DNA intercalator "complex"
   c. External sphere complex formation (Ionic binding)
   d. Internal sphere complex formation (Covalent binding)

3. BINDING OF cis-AND trans-DDP WITH DNA AND/OR PROTEINS
   a. Introduction
   b. Intrastrand Crosslinks (IntraSCL)
   c. Interstrand Crosslinks (InterSCL)
   d. 1,3-IntraSCL
   e. Bidentate binding of cis-DDP with one base of DNA
   f. Interaction of cis-and trans-DDP with proteins and formation of DNA-Pt-protein crosslinks.

4. ELEMENTS OF PHARMAKOKINETICS OF cis-DDP, PARAPLATIN AND IPROPLATIN

5. CONCLUDING REMARKS

### 1. HISTORICAL BACKROUND

(a) Introduction : The compound dichloro-diamine-platinum (II), of the empirical formula $Pt(NH_3)_2Cl_2$ was known since 1844 in two forms, the α-$Pt(NH_3)_2Cl_2$ or Peyrone salt (cis-platin) and the β-$Pt(NH_3)_2Cl_2$ or Reiset salt (trans-platin). The pioneering work of Werner [1] on the structure and bonding of coordination compounds, led to the assignment of a square planar geometry for these complexes (Fig. 1).

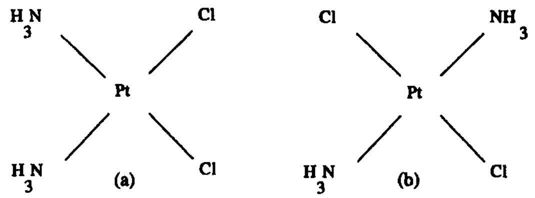

Fig. 1. The structure of (a) cis-platin or cis-DDP and (b) of trans-platin or trans-DDP.

The structure of both isomers was elucidated by X-ray diffraction studies [2].

Platinum (II) is classsified as a "soft" Lewis acid, according to Pearson [3] or class b, and generally forms more stable bonds with donor atoms characterized also as "soft" Lewis bases (e.g. N,S,P etc), while with "hard" bases (e.g. Cl, $H_2O$, $NO_3^-$, $CO_3^{2-}$, $SO_4^{2-}$ etc), it forms weaker bonds.

Studies on ligand substitution reactions in square planar complexes led to the development of the kinetic trans-effect by Chatt [4]. The discovery of trans-effect was made by the Russsian chemist Chernyaev [5]. It was subsequently studied in depth by Basolo [6], Drago [7], Sadler [8] etc and it continues to be studied even today.

Around 1965 the first publication appeared by B. Rosenberg and his collaborators [9] describing the unusual behavior of E.coli bacteria culture, in the presence of platinum electrodes. This was a part of a biophysical study of the growth of bacteria under the influence of the electric current. The bacteria were forming long filaments, about 300 times longer than the normal size [9]. This filamentous growth meant inhibition of cell division, but not of cell growth. The platinum electrodes were selected because of the inertness of the metal, while the observed filamentous growth was starting 1-2 hrs after the passage of the electric current. It was subsequently found that for this abnormal growth of the bacteria, small amounts of metal in the form of the complex $(NH_4)_2PtCl_4$ (1-10 ppm) were necessary. The same behavior was also observed in the presence of similar amounts (about 8 ppm) of complexes of other transition metals as well, like Rh, Ir, Ru, etc, with a more pronounced effect observed with Rh complexes, e.g. $RhCl_3$ or $(NH_4)_2RhCl_6$ [10].

Later it was found by Rosenberg etal. [10,11] that the platinum electrode formed electrolytically $[PtCl_6]^{2-}$, which after reaction with $NH_4Cl$, used as a nutrient in the medium in which E.coli bacteria were growing, was transformed into cis-$Pt(NH_3)_2Cl_4$. This in turn after UV

irradiation was producing among others cis-$Pt(NH_3)_2Cl_2$. Both cis-$(NH_3)_2PtCl_4$ and cis-$(NH_3)PtCl_2$ were the active species for the production of the filamentous growth. It was also observed that the trans-$Pt(NH_3)_2Cl_2$ analog was inactive.

Based on the above properties of the two cis-complexes of $Pt^{2+}$ and $Pt^{4+}$ and knowing since 1931 that ruthenium compounds and $RhCl_3$ were inhibiting the growth of adenocarcinoma and of mice sarcoma, Rosenberg [12] decided to test them against sarcoma-180 and mice lymphocytic leukemia 1210 and obtained the first satisfactory results. In 1973 Rosenberg [13] presented the results of clinical trials (phase I and phase II) for cis-$(NH_3)_2PtCl_2$ (cis-platin, or cis-DDP) started in 1972, as well as in vitro tests of various platinum complexes against a broad spectrum of tumors. Thus, from a broad spectrum of 28 tumor types with 50 to 100% cures (phase I clinical trials), 7 types of toxicity effects were discovered, with as more serious ones, nephrotoxicity, nausea and vomiting. Nephrotoxicity was determining the limiting dose of the drug. The best results of cis-DDP were found against testicular and ovarian cancers. The problem of nephrotoxicity on the other hand, was solved by the idea of Cvitkovic and Krakoff at the Sloan Kettering Institute, to use extensive hydration and mannitol prior to drug administration. Cis-DDP was subsequently used in combinational therapy with vinblastine and bleomycine against testicular cancer with >95% cures.

Analogs of cis-DDP exhibiting antitumor activity were also presented by Rosenberg in 1978 [14] (Fig. 2).

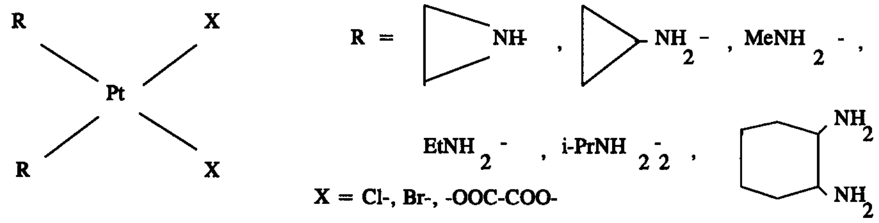

Fig. 2. Analogs of cis-DDP with antitumor activity.

Finally, in 1978 the complex compound cis-$(NH_3)_2PtCl_2$ (Peyrone's Salt) was approved for clinical use as a drug under the name "Platinol" by the F.D.A. (Food and Drug Administration) of the USA, and under the name "Neoplatin" in Great Britain in 1979 by the DHSS (Department of Health System and Security).

Since 1983 the drug comes first in sales in the USA and worldwide among all the analogous anticancer drugs. Today only the derivative of cis-DDP, cis-[$(NH_3)_2$Pt(CBDCA)], or cis-diamino-[1,1-cyclobutane-dicarboxylate-(2)-O,O'] platinum (II), named Paraplatin or Carboplatin, or JM-8 is commercialy widely used (Fig. 3). Figure 3 presents a few analogs of cis-DDP with satisfactory antitumor activity that are presently under clinical trials in various phases, except carboplatin, which is already in use. Carboplatin is used today in combination with other antitumor drugs

[15] or with radiotherapy against testicular, ovarian, head and neck cancers. It has however little effect against cancers with a great percentage of mortality, like lung cancer and cancers of the gastrointestinal tract [16].

Today a great deal of research work is still carried out around cis- and trans-DDP and their derivatives. In particular about 2000 relevant publications were referred, during the years 1987-1992 in the Chemical

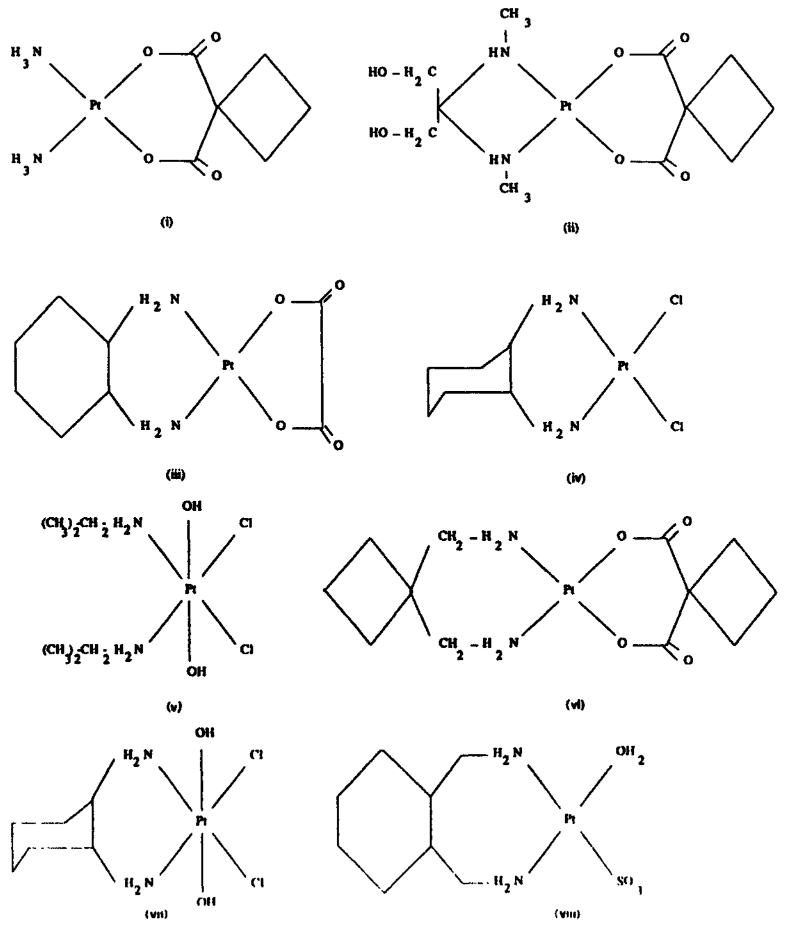

Fig. 3. Some newer antitumor complexes of Pt(II) and Pt(IV);
(i) Carboplatin or Paraplatin or JM-8 or CBDCA or cis-diamino-[O,O'-1,1-cyclo-butane-dicarboxylato] platinum (II), (ii) Zeniplatin or CL 286, 588 or [N,N'-2,2-bis(aminomethyl)-1,3-propanodiole] [O,O'-1.1-cyclobutane-di-carboxylato] platinum (II), (iii) Oxaliplatin (Oxalatoplatinum) or cis-(N,N-1,2-diaminocyclohexane)-(trans-I)-oxalato-platinum (II), (iv) DACH-Pt or cis-(N,N-1,2-diaminocyclohexane)-(trans-I)-dichloro-platinum (II), (v) CHIP or Iproplatin or cis-(bis-isopropylamine)-(trans-I)-dihydroxy-cis-dichloro-platinum (IV), (vi) Enloplatin or CL 287,110 or [O,O'- 1,1-cyclo-butane-dicarboxylato] [N,N-tetrahydro-4H-pyrane-4,4-dimethanamine] platinum (II), (vii) Tetraplatin (Ormaplatin) or NSC 363812 or Tetrachloro-[N,N-(d,1-trans)-1,2-diaminocyclohexane] platinum (II) and (viii) Spiro-platin or TNO-6 or [N,N-(1,1-bis-aminomethyl)-cyclohexane] (Sulfato)(hydrato)platinum (II).

Abstracts [17]. In 1984 Rosenberg was honored with the Charles F. Kettering award, sponsored by General Motors, where he presented a review of his work [18]. The present paper is attempting to briefly review what is known today on the mechanism of action of cis-DDP and carboplatin, which almost replaced the former and is the only one in use.

(b) Structure-Activity Relationship : The aquired knowledge on the activity and chemistry of cis-DDP and its inactive congener trans-DDP, even from the early studies and the testing of many analogs of the drug against various tumors, provided the first elements of structure-activity relationship of these complexes [19-26], summarized in the folllowing rules:
(i) The compounds should be neutral and correspond to formulae cis-$Pt^{II}(am)_2X_2$ or cis,cis,trans-$Pt^{IV}(am)_2X_2Y_2$, with am=amine and X,Y=anionic ligands (good leaving groups), (ii) The ligands X, should consist from groups or donor atoms that make bonds with Pt(II) of intermediate strength. For this or other reasons (e.g. activation by enzymes) these are good leaving groups. Examples are $SO_4^{2-}$, $Cl^-$, $C_2O_4^{2-}$, citrates $[(OOCCH_2)_2C(OH)(CH_2COO)]^{3-}$, or other dicarboxylates. For the compounds of Pt(IV), the ligands Y are mainly $OH^-$ occupying the axial trans positions. (iii) The amine ligands should have at least one N-H group whether they are mono or bidentate so that they will be able to form at least one hydrogen bond. All the compounds with nitrogen donor ligands containing no hydrogens capable to form hydrogen bonds, do not possess antitumor activity, though they can also bind with DNA.

The possible roles of the N-H group in the biological activity of the compounds, may either be kinetic (e.g. a possible relationship with the approaching of the macromolecule of DNA) or thermodynamic (e.g. with the additional (de)stabilization after binding with the biological target of DNA) or steric (e.g. hindered rotation, due to the increase of bulkiness of of the nitrogen donor ligand) or reasons related to the transport of the drug within the cell. In any case, it is even today a subject of extensive research [27].

It should be emphasized that the above rules are not universal and that some Pt(II) compounds with increased cytotoxicity or even antitumor activity do not obey them [28]. A few examples are given below:

(i) Complexes with two diamine units bridged with a carbonate bridge of formula $[\{cis\text{-}Pt(NH_3)_2Cl_2\}_2\{\mu\text{-}N,N\text{-}(NH_2(CH_2)_nNH_2)\}]$, [29-31] with maximum activity when n=5.

(ii) Dimeric or trimeric hydroxo-bridged complexes of Pt (II) of formulae, $[cis\text{-}[Pt(NH_2CH(CH_3)_2)_2\text{-}(\mu\text{-}OH)]_2(NO_3)_2$ and $[cis\text{-}Pt(R,R\text{-}DACH)(\mu\text{-}OH)]_3(NO_3)_3$, where DACH=1,2-diaminocyclohexane [32].

(iii) Complexes of formula $[Pt(diam)(RR'SO)Cl](NO_3)$, with diam=a bidentate amine and RR'SO=a sulfoxide [33].

(iv) Increased cytotoxicity was observed in complexes of type trans-$[PtCl_2(RR'SO)(quin)]$ and trans-$[PtL_2Cl_2]$, where, quin=quinoline and L=pyridine, N-methyl-imidazole, thiazole, quinoline [34-36].

(v) Compounds of formula cis-$[Pt(NH_3)_2(N\text{-}Het)Cl]^+$, with N-Het=a heterocyclic amine [37, 38] containing only one leaving group.

(vi) Antitumor compounds of formulae, $[Pt^{II}(N,N\text{-}NRCH_2CH_2NR)L_2]$ and $[Pt(N,N\text{-}NRCH_2CH_2NR')(py)_2]$, R=R'=polyphenyl, L=pyridine or methyl pyridine [39].

(vii) The trans-[$PtCl_2${E,E-HN=C(OR')R}$_2$] possesses a remarkable antitumor activity, larger than its cis-EE and cis-ZZ isomers [40, 41].

In conclusion, complexes with antitumor properties include cationic, compounds containing four nitrogen donor atoms around platinum, nitrogen donor ligands without hydrogens for hydrogen bonds, and complexes with trans geometry.

(c) Binding sites of cis-DDP responsible for cytotoxicity and side toxicities : From the beginning it was obvious that the understanding of the way that cis-DDP exhibits its antitumor action, i.e. the target molecule and the binding sites in the cell causing the antitumor action, was of great importance for the development of new more effective analogs with reduced toxicity.

It was soon found that DNA was the target molecule of cis-DDP within the tumor cells in vivo, because the drug had the following biological effects: (i) the production of a filamentous growth in bacteria, (ii) the production of lysogenicity, (iii) carcinogenicity, (iv) inactivation of viruses and (v) the inactivation of bacteriophage viruses that contain and transport DNA.

Cis-DDP had also the following biochemical effects supporting the above conclusion: (i) selective inhibition of the synthesis of DNA and not of RNA or proteins, (ii) inactivation of the substrate of the enzyme DNA-polymerase, (iii) selective binding with DNA, (iv) other analogous complexes are bound in a similar manner to cis-DDP with DNA and finally, (v) the relationship between the antitumor activity and inhibition of the growth of bacteria [42].

The fact that the nuclear DNA was the primary target of cis-DDP within the cell, led to extensive research of the study of the interaction of the drug and other platinum salts with DNA and its constituents in vitro with the aim to discover the binding sites of the metal, its general reactivity and the possible modifications of DNA caused by the binding, leading to death of the cell. In 1971, the first publication on the interaction of cis-DDP with DNA, studied with UV and CD spectroscopies appeared [43]. The hypochromicity shown in the UV spectra of the double helix of DNA, indicates perturbation on the structure of the macromolecule upon platinum binding, i.e., the disappearance of the stacking interactions between the bases (increase of $\varepsilon$). This was interpreted as rather due to a covalent bonding of Pt(II) with the bases of DNA. At almost the same time the ir spectrum of the complex cis-$Pt(ado)_2Cl_2$ (ado=adenosine) was studied in the solid state, with Pt(II) bound at the N7 atom of the base [44].

Various techniques were employed in subsequent studies of interaction of platinum salts with nucleosides and nucleotides (DNA constituents), leading to the conclusions that the metal could mainly bind at the N7 and N1 sites of purines and the N3 site of pyrimidines [45-53]. Later similar studies of nucleobases showed additional binding sites the N3 and O6 of purines and the exocyclic amino group ($-NH_2$) of pyrimidines [54, 55] (Fig. 4).

(d) Hydrolysis of cis- and trans-DDP in aqueous solutions : In aqueous solutions the two isomers, cis- and trans-$(NH_3)_2PtCl_2$ are hydrolyzed as shown in Fig.5 and Fig.6 [56-61]. The concentration of the various species formed depends in each case on the pH and the ionic strength of the solution. $^{195}Pt$ and $^{15}N$-NMR [60, 62, 63] have been used in many cases to identify the species formed, while many hydroxo-complexes were isolated in the solid state and their structure was solved by X-ray diffraction [17, 64-66].

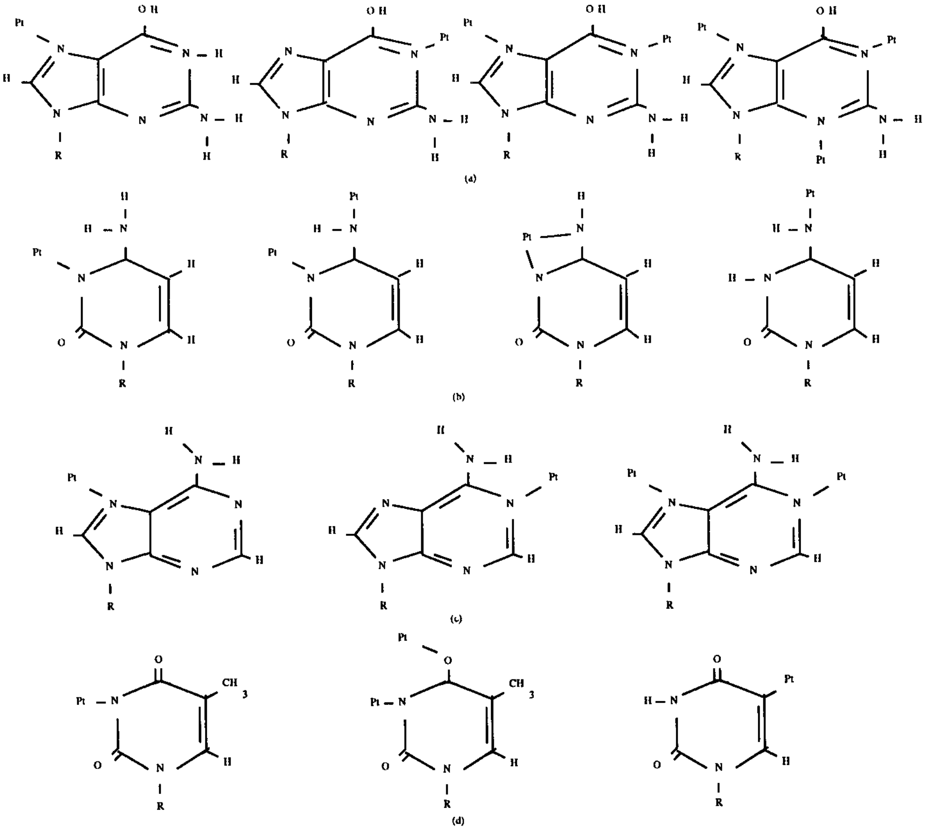

Fig. 4. Structures and bonding sites of (a) 9-Alkyl-Guanine, (b) 1-Alkyl-Cytosine, (c) 9-Alkyl-Adenine, (d) 1-Alkyl-Uracil and 1-Alkyl-Thymine.

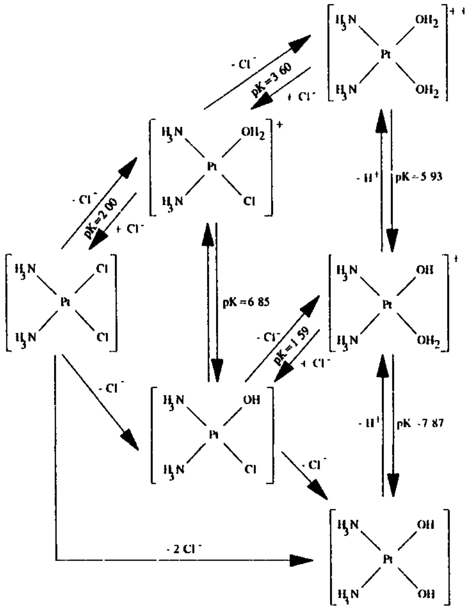

Fig. 5. Hydrolysis of cis-DDP in aqueous solution.

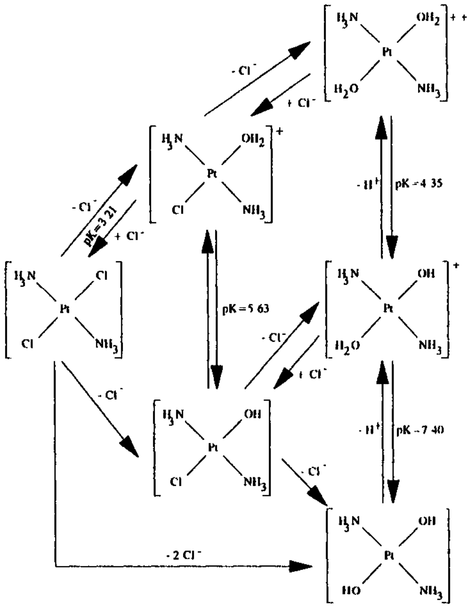

Fig. 6. Hydrolysis of trans-DDP in aqueous solution.

From Figs. 5 and 6, it is seen that the concentrations of the active monohydro-monochloro-diamine complexes in aqueous solutions, with ionic strength I=0.1 and temperature $25^{o}$, are 88% for the cis- and 54% for the trans-analog, calculated as percentages of the starting compounds.

The lower activity of trans-DDP compared to cis-DDP may be explained by the smaller value of pka of the former (5.63 compared to 6.85 for the cis-analog), for the equilibrium,

$$[(NH_3)_2Pt(H_2O)Cl]^+ \longrightarrow [(NH_3)_2Pt(OH)Cl] + H^+$$

As a result, under physiological conditions and since the $OH^-$ group is not a good leaving group as compared to $H_2O$, the concentration of the trans- mono-hydro-complex available is considerably reduced, consequently, its reaction with DNA is also reduced [56].

The blood plasma has pH=7.4 with chloride concentration of 100mM, while within the cell it is only 4 mM. Consequently, the hydrolysis reactions take place in the cell plasma, by the hydrated species with the proper atoms of the biomolecules. It is noted that Pt(II) is hydrolyzed faster than Pt(IV) and that the ligand substitution reactions in the former take place through a pentacoordinated intermediate [57].

It should also be noted that the species distribution of Figs. 5,6 is not strictly followed in vivo, since it is possible for the complexes to react with the various biomolecules, e.g. proteins of blood serum, in sulfur containing sites (glutathione, methionine etc) [67-70], prior to reaching thermodynamic equilibrium.

The hydroxo bridged species, that may also be formed in regions with high platinum concentration [71], are inert and believed to be related to the neurotoxicity of the drug. The release of ammonia from cis-DDP when the drug reacts with sulfur donors, like methionine [72], rises the question whether this release contributes to its cytotoxicity [72, 73].

### 2. BINDING MODES OF METAL IONS WITH DNA

(a) Introduction (Description of DNA structures) : The secondary structure of the B-form of DNA is shown in Fig. 7. Part of its primary structure with the possible sites on the bases for coordination with platinum is also given in Fig. 8.

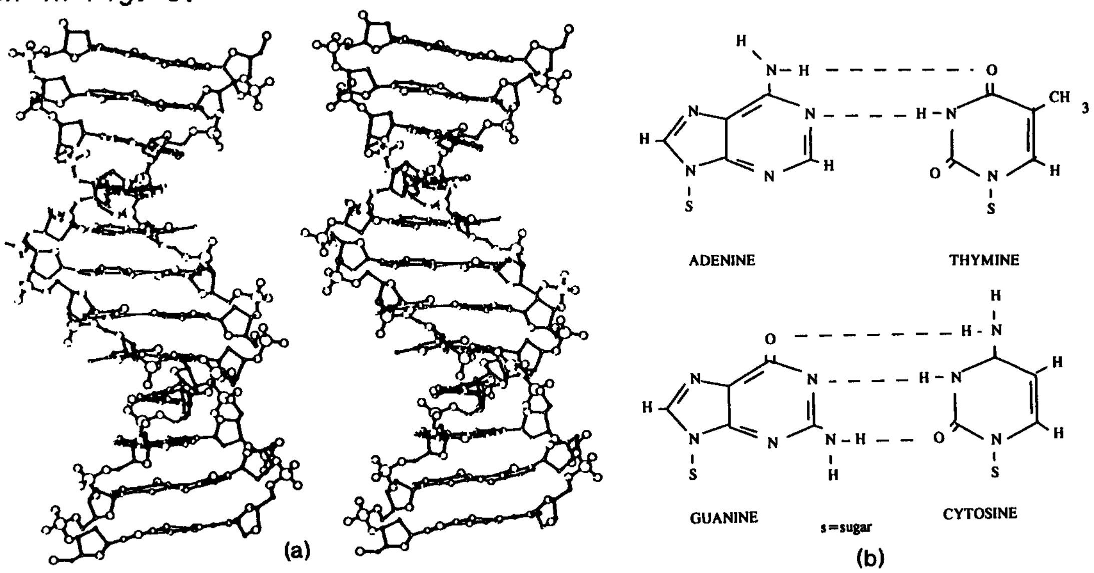

Fig. 7. (a) Structure of B-DNA ( Reproduced by permission from ref.[74] ) and (b) hydrogen bonds between base pairs, according to Watson-Crick.

Usefull parameters of the DNA structure [74] are,

(i) Translation per residue: The distance in the DNA axis, between two successive base pairs (in B-DNA it is 340 pm, or 3.4 Å).

(ii) Base pairs per turn: It is a measure of the magnitude of the helix and represents the total number of bases after a complete turn of the helix, i.e. after arriving again to the origin where it was started from (For B-DNA we have 10 pairs per turn, i.e., per 10 X 340 = 3400 pm).

(iii) Helical pitch : Distance run from the origin of the helix, required for a complete turn (in B-DNA is 3400 pm or 34 Å).

(iv) Base tilt : Angle of the plane of base pair with the perpendicular to the axis of the helix plane, at the same point. (In B-DNA it is $0^o$, i.e., every base pair determines a plane perpendicular to the helix axis or otherwise the planes determined by the bases are perpendicular to the axis).

(v) Base displacement : Distance of the centre of the base pairs from the helix axis (0 pm in B-DNA).

The B-form is the most common in solution. There are also the right hand side A-helix, that has a total number of 11 base pairs per turn, base tilt of $20^o$ and the left hand side, Z-form (zig-zag type), which is more scarce. On a B-helix, the axis of every base pair (i.e., the straight line connecting the centre of the bases) is turned in relation to the previous pair by $36^o$ (e.g. the angle of the two axes is $36^o$). In this way a major groove and a minor groove are finally formed in the macromolecule. The first (major groove) is 24 Å wide and it is considered as the primary recognition site of the proteins, while the second (minor groove) is only 10 Å wide with smaller depth and interacting only with small proteins. In the B-helix the minimum distance for two bases belonging to two different helixes is 7 Å [75].

(b) Formation of a DNA-intercalator "complex" : Intercalators are planar molecules, mainly organic polyaromatic systems, having the ability to intercalate between two successive base pairs kept near, by stacking interactions. A DNA-intercalator "complex" is thus formed. This possiblity of course exists only for a ds DNA and not for ss DNA. The effect of intercalation in the geometry of the molecule is the lengthening of the helix [76], so that the distance between the base pairs in the two sites of the intercalator is increased to 7-8 Å from 3,4 Å initially, resulting to biochemical changes, e.g. inhibition of enzymes like RNA polymerase and/or topoisomerase II [75].

Several structures of such complexes were solved with X-ray diffraction [77,78]. Proper experimental methods for the detection of this type of interaction, were developed based on changes of the structure of the macromolecule [79-81]. The structure of the complex $[(trpy)Pt(SCH_2CH_2OH)]^+$ (trpy:tripyridyl) with the dinucleotide dCpG (Fig. 9), which opens up the double helix of B-DNA by $23^o$ [82] or the structure of $[(trpy)PtCl]^+$ with AMP [78] are among the most characteristic ones.

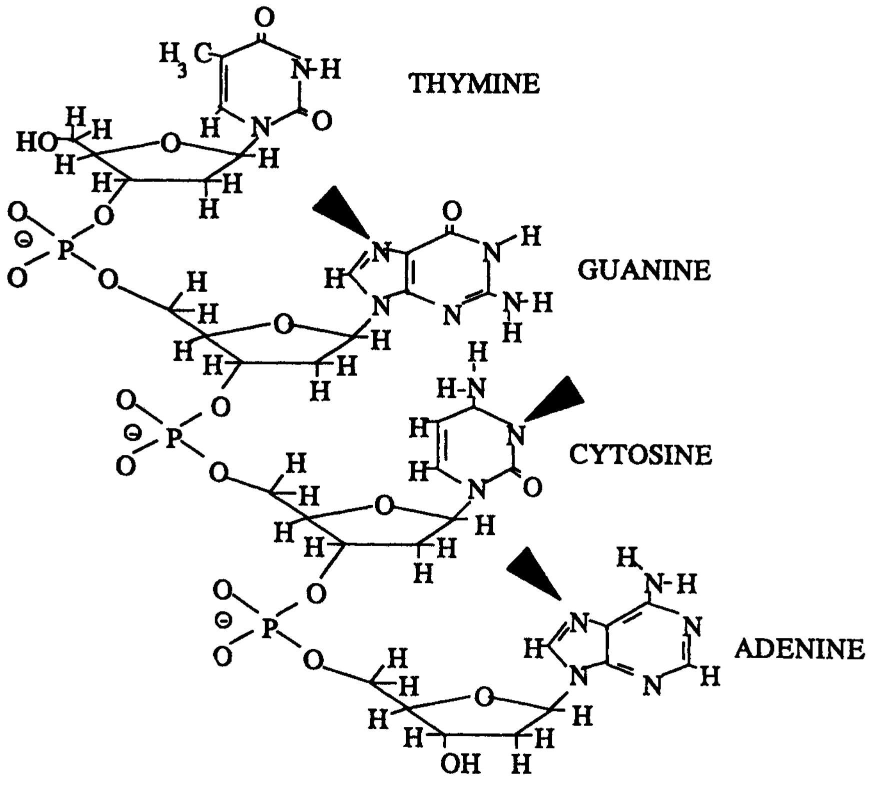

Fig. 8. The structure of the tetranucleotide model of a part of single helix (primary structure) of B-DNA, d(TpGpCpA), where the bonding sites with platinum are indicated.

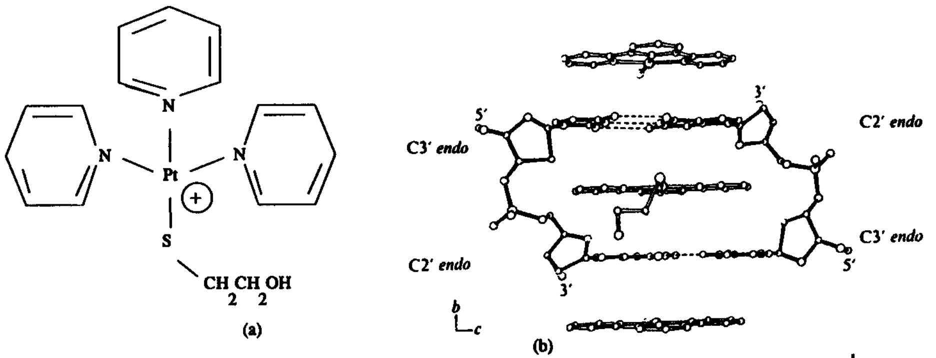

Fig. 9. (a) Structure of the complex $[(trpy)Pt(S\text{-}CH_2CH_2OH)]^+$ and (b) structure of its intercalator complex with the dinucleotide d(CpG) ( Reproduced by permission from ref.[82] ).

Others include the complex $[(trpy)PtCl]^+$ which reacts with regions rich in G-C of ds DNA and is used for selective protein labeling, since it has a great affinity for cysteine, much smaller for histidine and not at all for methionine [83], also of the "complex" $[(en)Pt(bpy)]^{2+}$ (en=ethylenediamine, bpy=bipyridyl) with AMP [84,85]. The interaction between these two molecules in solution was also studied by CD and $^1H$ NMR [86]. The angle of unwinding of DNA in these complexes was found to be 6-19$^o$, depending also on the presence of a coordination binding [87].

Finally, the so called "bis-intercalators" should also be mentioned. For example, in the complex $[\{(trpy)\}Pt\}_2(can)]^{3+}$ (can=canavarine) (Fig. 10), the aminoacid (canavarine) bridges two Pt(II) atoms as a "bis - intercalator", most probably through the minor groove of DNA [88]. Several analogs to these complexes with peptide bridges were also synthesized, in searching for compounds with selective binding with DNA [89].

Also, complexes of the type $\Lambda,\Delta\text{-}[M(phen)_3]^{n+}$ (o-phen=1,10-phenanthroline, n=2,3, M=Zn, Ru, Cu, Co, etc) may react similarly. With metals with more than one stable oxidation states like $Co^{3+}$, free radicals may be produced near their binding region with DNA, resulting to oxidative breaking of the macromolecule [42].

(c) <u>External sphere complex formation (Ionic binding)</u> : The counter ions of the negative charges of the phosphate groups in ds DNA that partially neutralize its charge are usually $Na^+$, or other small metal cations. They may of course be the positive parts of other biomolecules, like proteins. For example Au(III), in the form of $AuCl_4^-$ at low concentrations is bound to the phosphate oxygens, while at higher concentrations with the nucleobases [42]. Also, the complex $[Co(NH_3)_6]^{3+}$ or the $Mg(H_2O)_6^{2+}$ are known to stabilize the Z-form of DNA, through a B->Z transition [90]. Greater stabilization is achieved with the first complex, because (a) it has a larger charge, (b) it contains ligands forming hydrogen bonds and (c) ligands that are not readily exchanged with solvent molecules.

It should also be noted that the complex $[Co(NH_3)_6]^{3+}$ is widely used today as a reagent for the B⟶Z transformation of DNA with better results than the previously used methods for this purpose, i.e., high salt concentration (≈2.5 M $Na^+$) or ethanol (60%) etc.

Concerning the binding of platinum salts with DNA in this manner, molecular mechanics calculations showed that cis-DDP may interact either directly or through water molecules with the phosphate groups of the macromolecule [91,92].

(d) Internal sphere complex formation (Covalent binding) : Many metal ions are covalently bound with the bases of DNA. Extensive reviews on the subject exist [49, 51, 53], together with X-ray crystal structures [50, 93] and formation constants determinations in solution [94]. (See Section 1c, for conclusions).

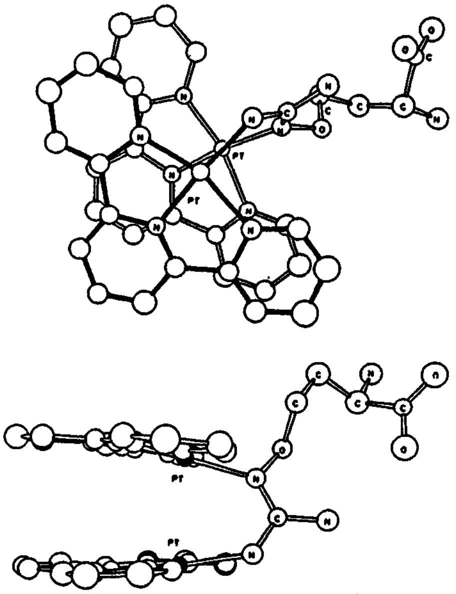

Fig.10. Molecular structure of the intercalator compound $\{[Pt(trpy)]_2Can\}^{3+}$ ( Reproduced by permission from ref.[88] ).

### 3. BINDING OF cis- AND trans-DDP WITH DNA AND/OR PROTEINS.

(a) Introduction : Both cis- and trans-DDP form covalent bonds with the donor atoms of the DNA bases, [26, 95-97]. The various ways of DNA binding of cis-DDP are given in Fig. 11.

More particularly, cis-DDP can bind with DNA in one of the following ways (Fig. 11).

(i) Monodentate binding with only one base of DNA.

(ii) Formation of "interstrand crosslinks" (InterSCL) with two bases belonging to different strands of DNA

(iii) Formation of "intrastrand crosslinks" (IntraSCL) with two successive, similar or not, bases of the same strand of DNA

(iv) Formation of DNA-Pt-protein crosslinks, and

(v) Bidentate binding with only one DNA base.

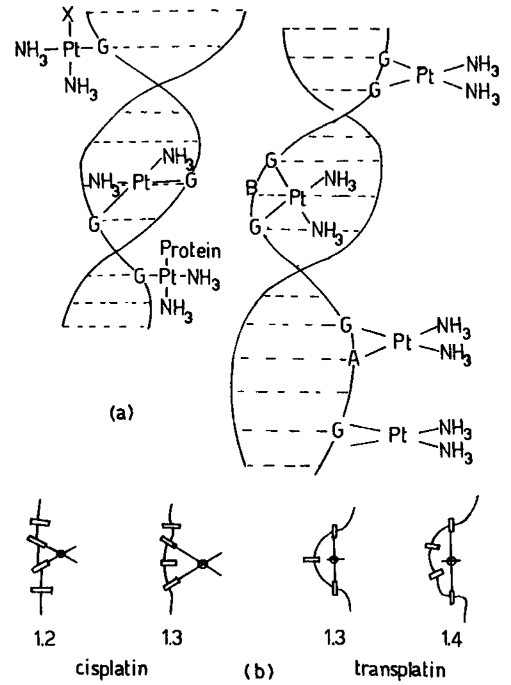

Fig. 11. Various ways of (a) DNA binding of cis-DDP or/and proteins and (b) differences in the conformation of the macromolecule from the formation of 1,2- or 1,3-IntraSCL by cis-DDP and 1,3- or 1,4-IntraSCL by trans-DDP.

The percentage formation of each type of bonding were quantitatively calculated [98,99,100]. Thus the percentage of the intrastrand crosslink betweeen two successive bases of guanine of the same strand of DNA (1,2-IntraSCL) formed by cis-DDP on the d(GpG) part of the macromolecule, varies from 50-60%, whereas IntraSCL between successive guanine and adenine bases (d(ApG)type) by 20-30% percentage. About 10% of cis-DDP forms 1,3-InstraSCL with two guanine bases separated by a third base of the type d(GpXpG). More scarce are the InterSCL between guanine bases belonging to different strands of DNA (<1%) and the DNA-protein crosslinks (<1%). More types of binding are also taking place in small percentages, but they have not yet been identified [98,99].

(b) Intrastand Crosslinks (IntraSCL) : The reactions of cis- and trans-DDP with DNA proceed in various steps, where kinetic factors play an important role. The rate determining step of the monodentate binding of the two isomers with DNA, is the substitution of a chloride by a water molecule, with half life $t_{1/2}$=1.9 hrs for trans- and 2 hrs for cis-DDP [101]. The monohydrated species produced, react fast mainly with the N7 of guanine of DNA, which is not only nucleophilic but also easy to be reached by the salt, in the direction of the major groove in B-DNA. In a first stage, electrostatic interactions between the negatively charged sites of the macromolecule (e.g. phosphates) and the cationic monohydrated species $[(NH_3)_2Pt(H_2O)Cl]^+$ seem to be important. According to our knowledge, cis-DDP prefers mainly parts of DNA containing two successive guanine bases, e.g. of the d(GpG) type, but not all of them are equally possible for attack [102]. The hydrolysis of the second chloride is again the rate determining step, $t_{1/2}$=2.1 hrs for the cis- and $t_{1/2}$=3.1 hrs for the trans-isomer, at pH=6.5 [101].

Experimental evidence for the formation of intraSCL in DNA between two N7 atoms of neighboring G molecules, also referred as a 1,2-IntraSCL, are provided by the following series of experiments:

(i) A large increase is observed in the value of the flotation density for the polynucleotide, poly(dG)poly(dC) that was treated with cis-DDP, compared to the corresponding increase of the platinated poly(dG.dG) and the platinated monomers.

Both poly(dG.dC) and poly(dG).poly(dC) are synthetic polymers with known base sequences. Poly(dG.dC) contains G:C=1:1 in a strict sequence and it has a double helix structure with the guanine bases taking the anti-conformation.

The poly(dG).poly(dC) is a high M.W homopolymer of double helix structure and it contains 43% dG and 57% dC. The first helix is poly(dG) and the other poly(dC), with the sugar in the former adopting a syn-conformation [103].

The fact that only poly(dG).poly(dC) contains adjacent guanine bases, shows the importance of the 1,2- IntraSCL (large increase upon platination in the flotation density value). Trans-DDP increases the flotation density of DNA proportionally to its G-C content, but it does not increase it so much for poly(dG).poly(dC), in contrast to cis-DDP, i.e., it is not showing any selectivity in the formation of IntraSCL, in neighboring bases [104].

(ii) The breaking of platinated DNA with enzymes possessing selectivity, e.g., restriction endonucleases. This selective breaking is inhibited by cis-DDP when the sites cut by the enzyme, have nearby the sequence, oligo (dG).oligo(dC) [105-107].

Also in experiments with exonucleases and with the use of techniques for sequence analysis of DNA, it is seen that the breaking of the platinated DNA is inhibited in oligo(dG) sites, most probably due to the formation of IntraSCL with cis-DDP [106-109].

(iii) NMR spectroscopic techniques on the interactions of di- and oligo-nucleotides with cis- and trans-DDP show the formation of IntraSCL.

(iv) Binding of the two isomers cis- and trans-DDP with DNA followed by hydrolysis (mainly enzymatic or other) and identification of the obtained products with chromatographic HPLC and FPLC analysis and $^{1}$HNMR, showed for cis-DDP the existence of only 1,2-IntraSCL of the GG and AG but not of the GA (5'⟶3') type [99, 110, 111].

Thus, $^{1}$HNMR and chromatographic methods of the reaction products of cis-DDP with various homo- and hetero- dinucleotides showed that there is a kinetic selectivity in the reaction with d(GpG), while the reaction with d(ApA) was very slow [112].

In the case of the reaction of cis-DDP with d(GpC), a 1:2 complex of the metal with the ligand is formed, bound only through $N_7$ of G. Cytosine is not taking part in the complexation, under the conditions of the reaction, showing the lack of selectivity for a 1,2-IntraSCL with d(GpC).

Studies on interactions of cis-DDP with the autocomplementary hexanucleotide d(ApGpGpCpCpT) , followed by HPLC chromatographic separation and characterization of the products with 2D-$^1$HNMR spectroscopy, showed the formation of almost exclusively of a 17-membered chelate ring or an 1,2-IntraSCL between the metal and G(2), G(3) through N7 [113, 114].

The same hexanucleotide reacting with trans-DDP produces mainly 1,3-IntraSCL, forming a 23-membered chelate ring, involving the N7 of A(1) and N7 of G(3) [115] (Fig. 12).

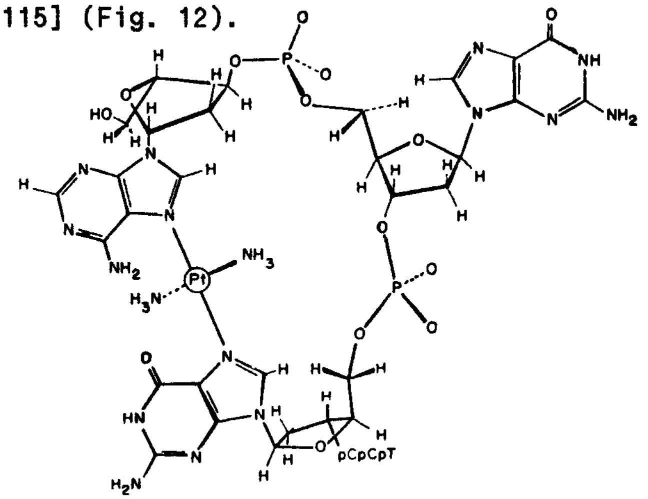

Fig. 12. Binding of trans-DDP with the nucleotide d(ApGpGpCpCpT)
( Reproduced by permission from ref.[115] ).

Trans-DDP on the other hand after reaction with DNA and enzyme hydrolysis separation of the products with HPLC and identification of them with atomic absorption and $^1$H NMR spectroscopies, formed intrastrand crosslinks with single stranded DNA of the dG-Pt-dG type (about 60%), of the dG-Pt-dA type (about 35%) and dG-Pt-dC (about 5%). With double stranded DNA the corresponding amounts were dG-Pt-dG (50%), dG-Pt-dA (40%) and dG-Pt-dC (10%). The differences have been attributed to the greater lability of the single than the double stranded DNA [116].

The difference between cis-DDP and trans-DDP seem to be related to their IntraSCL type that they form with DNA. While cis-DDP forms a chelate ring with two successive guanine bases (1,2-IntraSCL), trans-DDP seem to prefer a 1,3-IntraSCL with two non adjacent guanine bases of the type d(GpXpG) (X=any nucleobase). Both IntraSCL however, inhibit the synthesis of DNA, while a monodentate binding, e.g. of $[(dien)PtCl]^+$ (dien=diethylene triamine) does not [117].

The similar $t_{1/2}$ of the monodentate complexes with DNA of both isomers, show that the biological inactivity of trans-DDP cannot be due to the rate of the formation of a bidentate complex [116]. It can however be related to the reaction rate of sulfydryl (SH) groups with the monodentally bound to DNA trans-DDP, which is faster in comparison with the ones of cis-DDP, as it is seen with reactions of both with glutathione (GSH) [118].

Further studies with $^1H$ NMR [119] on the interaction of <u>cis</u>-DDP with the oligonucleotide d(TCTCGGTCTC)d(AGAGCCAGAG) show chelation with the two adjacent guanine bases at the center of the one strand. The results also showed that the double helix structure was present after platination [119].

Crystal structure determinations have also been carried out on some of the reaction products of <u>cis</u>- and <u>trans</u>-DDP with oligonucleotides. Very important was the elucidation of the crystal structure of the reaction product of <u>cis</u>-DDP with d(pGpG) forming a 1,2-IntraSCL [120,121] (Fig. 13). There are four crystallographically independent molecules in the unit cell. The guanine planes are in a head to head arrangement and their angle vary from 76.2$^o$ to 86.8$^o$ in each one of them. The sugars adopt the C3'-endo conformation.

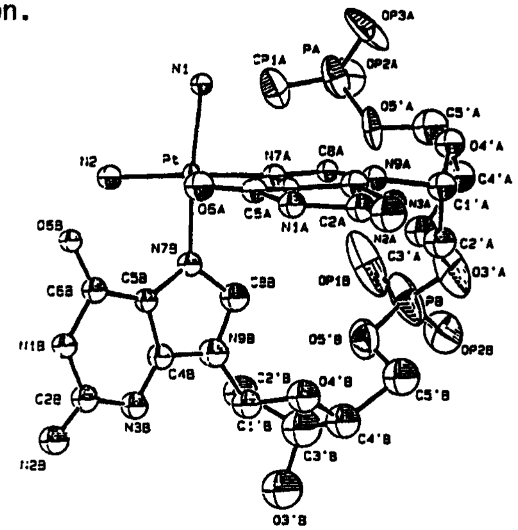

Fig. 13. Crystal strucure of the complex <u>cis</u>-[$(NH_3)_2$Pt($N_7$,$N_7$-d(pGpG)] ( Reproduced by permission from ref.[121] ).

This corresponds to the type of bonding that <u>cis</u>-DDP is expected to make in majority with DNA. It implies a bending of the double helix axis of DNA by 32-34$^o$. It can not be given by <u>trans</u>-DDP, since the distance between its two chlorines is 4.64 Å, compared to the 3.34 Å in <u>cis</u>-DDP, comparable to the distance of the two adjacent guanine bases in B-DNA [2,75].

The elucidation of this structure was confirming previous $^1H$ NMR studies on the interactions of <u>cis</u>-DDP with the d(GpG) forming 1,2-IntraSCL in solution [122].

Similar 1,2-IntraSCL was also found in the structure of the complex <u>cis</u>-[$(NH_3)_2$Pt(d(GpGpG)-$N_7$(2),$N_7$(3))] [123].

Other crystallographic studies include the structures of <u>cis</u>- and <u>trans</u>-DDP with t-$RNA^{phe}$ from brewer yeast with 6 and 3 Å resolution, respectively [124, 125]. <u>Cis</u>-DDP formed 1,2-IntraSCL of the type d(GpG) and d(ApG) [124], while <u>trans</u>-DDP [125] is monodentally bound at G(34) [125]. Also in the structure of [(dien)Pt[d(ApGpA)-$N_7$(2)], complexation takes place only through N7 of G(2) (Fig. 14).

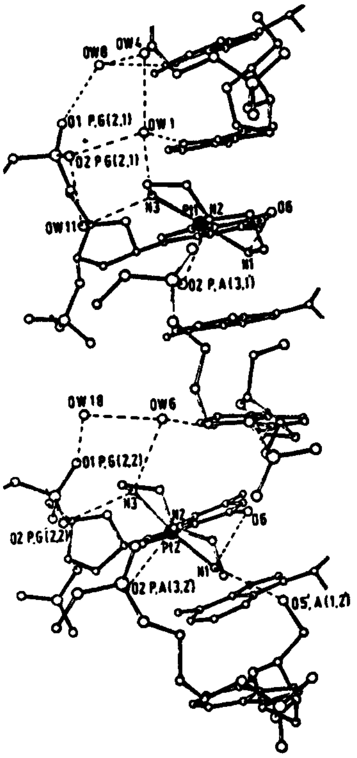

Fig. 14. Crystal structure of the complex (dien)Pt[$N_7$(2)-d(ApGpA)], with 1.15 Å resolution ( Reproduced by permission from ref.[123] ).

Molecular mechanics calculation studies [126] on the <u>cis</u>-DDP adduct of ds oligonucleotide of B-DNA type d(TCTCGGTCTC).d(GAGACCGAGA) containing mainly 1,2-IntraSCL with the N7 of G(5) and G(6) showed that two bending possibilities of the double helix axis in the major groove exist, with angles of $50^{o}$ and $60^{o}$. The angles of the two complexed bases are then $63.2^{o}$ and $58.4^{o}$ correspondingly [127]. The hydrogen bonding between the two complexed guanine bases with their complementary cytosines is also modified. The sugar conformation is changed to C3'-endo from C2'-endo for the platinated guanosines and a hydrogen bonding exist between the ammonia molecules of <u>cis</u>-DDP and the 5'-phosphate group. Of the two bending models for the structure of B-DNA in the presence of <u>cis</u>-DDP, the one corresponding to $63.2^{o}$ is more stable by 19.3 kcal/mol under high ionic strength conditions (i.e., with maximum neutralization of charge of DNA), called "model of high salt concentration", while in lower ionic strength the $58.4^{o}$ model is more stable, called "model of low salt concentration" [126].

Similar molecular mechanics calculations for the binding of <u>cis</u>-DDP with nucleotides of the type XpG and GpX, (X=base), show selectivity for platination when X=G [128].

The calculated bending angles of the double helix of DNA with molecular mechanics may however differ considerably from the real values, if factors like interactions with the solvent etc are not taken into account. For example, when the part of B-DNA containing 22 base pairs of the type (Scheme I), representing 2.5 turns of the helix, is treated

TCTCCTTCTTGGTTCTCTTCTC
AGAGGAAGAACCAAGAGAAGAG

Scheme I

with cis-DDP followed by gel electrophoresis and comparison with a part of DNA with bending angle of $20^o$, it is found that the bending angles in the former with 1,2-IntraSCL vary from $35\text{-}40^o$, differing considerably from the ones calculated by molecular mechanics of $50\text{-}61^o$ [129,130].

Combined molecular mechanics and 1D- and 2D- $^1$H NMR studies, on the reaction of cis-DDP with the double helix type 10-nucleotide d(GCCGGATCGC).d(GCGATCCGGC) showed a bending angle of the helix by $55\text{-}62^o$ after the formation of 1,2-IntraSCL of the metal with G(4) and G(5) and an unwinding of it by $12\text{-}19^o$ [131].

Recent studies on the cytotoxicity of the 1,2-IntraSCL if d(GpG) type caused by cis-DDP on the DNA of E.coli, using as a model the 12-nucleotide d(TCTAGGCCTTCT) [132], in combination with previous work on the cytotoxicity of the 1,2-IntraSCL of d(ApG) type [133] show that this latter type of binding should be about 5 times more toxic than the former one.

This indicates that we have to be cautious in determining the more cytotoxic IntraSCL of the drug.

During the replication of platinated DNA, the 5-position of the sugar presents the larger distortion, mainly due to the structural change to the C3'-endo conformation of the latter. As a consequence mutation may occur at this position (replacement of C by A) and not at the 3' where the distortion is smaller (maintenance of the C2'-endo configuration) [132] (Fig. 15).

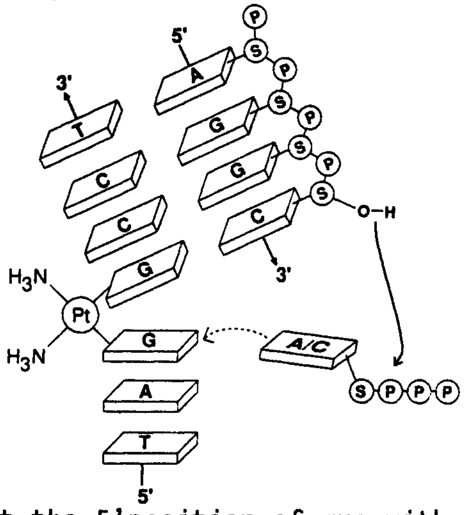

Fig. 15. Mutation at the 5'position of guo with C3'-endo sugar conformation (Reproduced by permission from ref.[132] ).

A comparison of the effects caused by platination (cis-DDP) and methylation at the N7 position of dGuo and dGpG, shows that the former stabilizes more than the latter the N-glycosidic bonding towards acid hydrolysis and does not cause the opening of the imidazole ring [134]. This means that the N7 platinated guanines are slower hydrolizing the N-glycosidic bonding than the methylated ones, e.g. slowly apopurinate from DNA. Also the difference from a nucleoside (dGuo) and a dinucleotide (GpG) upon platination consists to the easier breaking of the N-glycosidic bonding in the former and of the Pt-N7 bonding in the latter [134].

It was finally proposed that for the formation of the 1,2-IntraSCL at the d(GpG) section of DNA, the cis-DDP is initially accidentally bound with one guanine base monodentally through N7 in a reversible fashion. In this way, cis-DDP may "march" on the double helix until it is able to form a permanent 1,2-IntraSCL when it "finds" two adjacent guanine bases [135].

(c) Interstrand Crosslinks (InterSCL) : Usually the percentage of InterSCL is less than 1% of the amount of cis-DDP bound with DNA, though it seems to increase with time up to 3% [136] or even up to 5% according to others [137]. Both isomers cis- and trans-DDP form such bonds in comparable amounts [106].

It should be noted that the covalent binding of cis-DDP with the DNA bases creates local unwinding of the double helix, resulting to regions of single stranded structures within the macromolecule and to a final shorterning of the active length of it [97].

In DNA rich in G,C, it was proposed [138] that the initial formation of a $N_7O_6$ chelate with cis-DDP of guanine has as a result the breaking of the hydrogen bondings between the two bases G,C in opposite strands. This was followed by the formation of interstrand crosslinks between the deprotonated N1 atom of guanine and the N3 of cytosine [138]. This was however, never confirmed.

The formation of InterSCL between two guanine bases of opposite strands, bound to Pt(II) through their N7 atoms create an important distortion of the secondary structure of DNA, as molecular three dimensional models show. More particularly the two helixes are turned in opposite directions approaching each other and the distance between the glycosidic bonds is reduced to 10 Å from 12 Å.

It was also proposed that such InterSCL are formed between G-C(5'⟶3') pairs, though this was not confirmed [139].

When cis-DDP reacts with the oligonucleotide d(CTTCTCCTTGCCTCTCCTTCTTC) and after isolation and indentification of the products with gel electrophoresis and molecular models, it was found that the sequence d(GC/CG) which was platinated was distorted and a bending of 55° was observed in the direction of the major groove. The average angle of turn of the bases is kept constant however. This occurs, because for the formation of the InterSCL the two guanine bases should approach to 3.4 Å (bite distance in cis-DDP) from the 7 Å, which is their normal distance in B-DNA [140].

The InterSCL may not play a very important role in the cytotoxicity of cis- and trans-DDP [141] or of cis- and trans-diamino-tetrachloro-platinum (IV) [142], since it is only formed in a very small percentage, though more recent results show the opposite [143, 144].

(d) 1,3-IntraSCL : As already mentioned, both cis- and trans-DDP can form 1,3-IntraSCL. More details of the 1,3-IntraSCL formed by cis- and trans-DDP are given here.

$^1$H NMR studies on the interaction of the trinucleotide d(GpCpG) with trans-DDP showed complete unstacking of the bases, while the sugar of G(1) changes conformation from C2'-endo to C3'-endo[145]. This is similar to the change caused to the sugar at the 5'-position, in the 1,2-IntraSCL of cis-DDP with d(GpG), but differs from the 1,3-IntraSCL of cis-DDP with d(GpCpG) [146] favoring the C2'-endo conformation. This may be related to the lack of antitumor properties of trans-DDP.

Also the reaction of trans-DDP with the oligonucleotide d(CCTCGAGTCTCC) (12-bases) gave products that were characterized after enzymatic digestion and HPLC chromatographic separation, by $^1$H NMR spectrosopy [147]. The reaction had taken place at pH=3, so that complexation at N1 of A(6) (pK=3.8) was avoided and resulted in the formation of the 1,3-IntraSCL [147]. Monodentate intermediates of trans-DDP through the N7 of G(5) or G(7) can be detected.

When the double stranded 12-nucleotide d(CCTCGAGTGTCTCC).d(GGAGACTCGAGG) is used instead, it is seen from the molecular models of the free and platinated nucleotide (Fig. 16) that because of the 1,3-IntraSCL, the A(6) at the middle is not anymore stacked with the complexed guanine bases

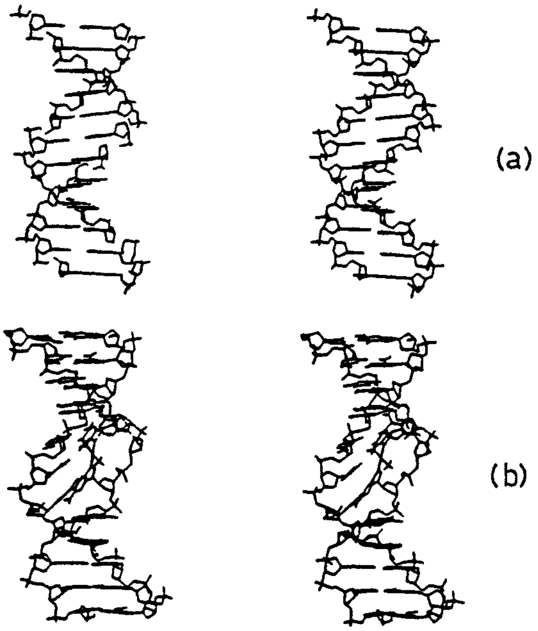

Fig. 16. Molecular models of (a) free and (b) bound with trans-DDP, polynucleotide d(CCTCGAGTTCTCC).d(GGAGCTCAGAGG) (Reproduced by permission from ref.[147] ).

surrounding it and its sugar takes the C3'-endo conformation [147]. The difference from cis-DDP [127] is due to the fact that this causes a considerable bending of the double helix in the main groove of B-DNA, by $40^o$-$70^o$, because of the 1,2-IntraSCL, while the trans-DDP bending angle is only $18^o$, because of the 1,3-IntraSCL.

The rate constant of the formation of the 1,3-IntraSCL, with the nucleotide of trans-DDP was, $k=(12.5\pm0.4) \times 10^{-5} sec^{-1}$, while with DNA had the comparable value of $k=(9.6\pm0.4) \times 10^{-5} sec^{-1}$ [115], as measured with $^{195}$Pt NMR spectroscopy.

Atomic absorption spectroscopy gave the value $k=(5.4\pm0.4) \times 10^{-5} sec^{-1}$ [115], for the rate constant of the reaction of DNA with trans-DDP [148], while it is reminded that the rate constant for the replacement of the first chlorine by a water molecule (Fig. 6) was $k_1'=1.9 \times 10^{-5} sec^{-1}$. This indicates that trans-DDP binds to DNA in two pseudofirst order steps, the first being the chlorine replacement and the formation of a monodentate complex with the N7 of guanine and the second, which is faster, the formation of the 17-membered chelate through the N7 atoms of G(5) and G(7) of the nucleotide [148]. The rate of the total reaction seems to be determined from the replacement of chlorine by water.

The formation of a bidentate complex by the two isomers was considered until today as non reversible. Recently however, it was found that during the reaction of the 12-nucleotide 5'-d(TCTACGCGTTCT) with trans-DDP at low pH and after enzymatic hydrolysis of the products and characterization with HPLC and $^1$H and $^{195}$Pt NMR spectroscopy, a 1,3-IntraSCL between the N7 of G(6) and G(8) was formed initially, followed by a rearrangement of a 1,4-IntraSCL between the N3 of C(5) and the N7 of G(8), in neutral aqueous solutions [149]. The equilibrium constant of this rearrangement reaction was found to be 3, with preference to the formation of 1,3-IntraSCL. The half life time of the 1,3-IntraSCL was 1.29 hrs at $30^oC$ and 3.6 hrs at $62^oC$, with $\Delta H^{\#}=91\pm2$ kj/mol and $\Delta S^{\#}=-58\pm8$ J/mol K [149].

In conclusion, the 1,3-IntraSCL type bonding formed by both cis- and trans-DDP does not seem to be responsible for the antitumor action of the former, although it partially inhibits the DNA synthesis. Also, the formation of the IntraSCL should not be considered as non reversible, like in the case of the 1,3⟶1,4 rearrangement, although the biological importance of the latter is not as yet understood and is under investigatioon [149].

(e) Bidentate binding of cis-DDP with one base of DNA : The formation of a N7O6 chelate of Pt(II) with the same base of guanine was proposed in the past, both with DNA [138, 150] and with simple guanosine [48] based on spectroscopic data and chemical reactions.

Similar chelates with Pd(II) were also proposed [151-153].

The importance of the possible formation of such a chelate by cis-DDP is immediately obvious, since trans-DDP is unable to form it. This might explain the antitumor properties of the former and the inactivity of the latter.

Various oxopurines can form such N7O6 chelates. For example, in the complex $[(\eta^5\text{-Cp})_2(\text{Theoph})\text{Ti}]$ [154], theophylline is bound bidentally through N7O6 with Ti(IV). Similar chelates were also found in the complexes $[(\eta^5\text{-Cp})_2\text{Ti}]_3(\text{HXan})]\text{Cl}$, (Xan=Xanthine) [155] and

$[(8\text{-MeS-Theoph})_2Cu(py)_2]$, (8-MeS-Theoph=8-methylthio-theophylline) [156]. The hexameric structure of the complex $[(CH_3)_3Pt\text{-}(theoph\text{-}2HCl)_3]_6$ contains also such chelates [157]. Also, when the complex mer, trans-$[(dien)Pt^{IV}(OH)_2(N7\text{-}9\text{-}MeG)](ClO_4)_2.2H_2O$ is left for one day at 50 $^oC$ with 2 equivalents of DCl, the mer-$[(dien)Pt(OH)Cl(9\text{-}MeGH\text{-}N7)]^{2+}$ is obtained. In the $^1H$ NMR, it is $\delta_{H(8)}$ = 8.37 ppm. After 12 more days new products with $\delta_{H(1)}$ = 8.50 and $\delta_{H(8)}$ = 8.54 ppm are observed, which may be due either to N7O6 chelates or N1, N7 bridged complexes [158].

Cytosine, a pyrimidine base, can also form a four membered chelate ring between the N3 atom of the ring and the exocyclic amino group at 4 [159]. Macrocycle chelates may also be formed between the N7 of guo and the phosphate group and cis-DDP in DNA as $^{31}P$ NMR studies showed. The phosphorous signal is shifted downfield by 1.4 ppm and this is not observed with trans-DDP [160]. This result was also more recently interpreted as due to a particular conformation of the oligonucleotides called hairpin-like [161, 162].

Indications for the formation of such bonds have also been given for the reaction of cis-DDP with GMP, with fast atom bombardement mass spectroscopic techniques, on the products of the reaction of 1:1 stoichiometry [163].

In conclusion, the N7O6 chelate of guanine or any other oxopurine with cis-DDP has never been found yet in any crystal structure solved with X-rays, that unequivocally would prove its existence. Therefore the formation of such a chelate *in vivo* seems unlikely [164].

(f) Interactions of cis- and trans-DDP with proteins and formation of DNA -Pt-protein crosslinks : Cis-DDP reacts with plasma proteins and about 50% of the drug is bound there one hour after administration [24]. The main binding site of the drug is the sulfur of glutathione [165-167] or the sulfydryl group of cysteine. This binding was correlated with the higher nephrotoxicity of the drug compared to Iproplatin and Paraplatin [167]. Due to the high trans-influence of Pt(II), ammonia liberation is observed in such reactions in the plasma, by $^{15}N$ NMR spectroscopy [72]. Trans-DDP is bound to a larger extent than cis-DDP by histone proteins [168], while cis-DDP reacts faster with non-histone proteins [169].

Both cis- and trans-DDP are bound to chromatine forming DNA-Pt-protein crosslinks or IntraSCL of proteins [170-172]. Trans-DDP reacts mainly with the S atoms of Cys, Meth, or with the nonprotonated His, at physiological pH [173-175].

More specifically, it was proposed that cis-DDP is forming selectively crosslinks between the low molecular weight and high mobility group proteins HMG1 and HMG2 and DNA and that the bonding with the latter takes place on the main groove, while the proteins retain an α-helix structure [175].

Crosslink bonds are also formed by both isomers with non-histone proteins of HeLa cells, though trans-DDP to a larger extent. Both isomers bind to a larger proportion than in histone proteins, due to the greater methionine content of the latter [176, 177]. Such crosslinks have been correlated with the antitumor or cytotoxic action of the drug [178-180]. Since their percentage is small however, this seems unlikely today [95].

Also, the DNA-Pt-protein crosslinks for cis-DDP are more resistant to repairing enzymes than those for trans-DDP [181]. Furthermore, the formation of more DNA-Pt-glutathione crosslinks of cis-DDP than of trans-DDP, was explained with the lower toxicity of the drug, since in this way it forms a smaller number of 1,3-IntraSCL with DNA [182].

Due to the easier formation of DNA-Pt-protein crosslinks by trans-DDP, the latter was used to map regions of t-RNA in the vicinity with a protein. For example, such a crosslink is formed between the $t\text{-}RNA^{val}$ of the yeast of Saccharomyces cereviase (G) and of the protein aminoacylo-t-RNA-nval--synthetase [183]. The sites of the crosslinks of trans-DDP are the ones of $t\text{-}RNA^{val}$ that strongly interact with the protein and in this way they are determined [183].

Proteins play an important role in the formation of a "complex" with DNA, once the IntraSCL of 1,2-type with cis-DDP are formed. For example, it is known that the tripeptide LysTrpLys form a stacking complex only with DNA treated with cis-DDP but not with trans-DDP. This was attributed to the formation of certain parts with primary structure in the macromolecule [184].

In 1990 Lippard et al [185] discovered a group of proteins, named DNA Damage Recognition Proteins-DRP's. These "recognize" i.e., form selective complexes only with the 1,2-d(GpG) and d(ApG) IntraSCL of cis-DDP with DNA and not with the 1,3-IntraSCL. They are also unable to recognize the corresponding 1,3-IntraSCL of trans-DDP [185].

The action of these proteins ($MW=1X10^5$) was correlated with the unwinding of the DNA double helix, which in the case of 1,2-IntraSCL is $13^o$, while in the case of the 1,3-IntraSCL with cis-DDP is $23^o$, also to the bending of the helix axis in the 1,2-IntraSCL, being $35^o$ [186].

Extensive work was done afterwards for determination of the structure of these proteins. It was found [187-190] that they belong to the group of the non-histone proteins and called High Mobility Group, HMG1,2. HMG1 and HMG2 consist of 214 and 209 aminoacids respectively with N-terminal aminoacid glycine and differ in 43 positions (about 20%), e.g. in 6, 33, 38, 56, 65, 71 etc. They are consisting of three parts; one very acidic (C-terminal part) involving almost exclusively aspartic and glutamic acids, while the two others are consistent of two homogenic parts of 80 aminoacids each.

The proteins have a spheric structure with a positively charged nucleus able to "bind" with DNA, through its negative charges, with electrostatic interactions [187]. They have the ability (i) to bind strongly with the primary rather than the secondary structure of DNA [191, 192], (ii) to destabilize or to unwind the DNA double helix [193], (iii) to promote superhelix formation of plasmids and (iv) to replace histone proteins when they "bind" with DNA [187].

It was further proposed [187, 189] that they are forming two α-helixes crossed in a "X" shape, which selectively also recognize DNA of crossed type. They are formed during the superhelix formation of natural DNA [187-189].

It is believed that the 1,2-IntraSCL of cis-DDP are protected by these proteins and in this way are not "recognized" by cell enzymes leading to the final death of the cell. This theory is under extensive investigation [190]. To this behavior of the proteins, the negative charge of their terminal carboxylate groups, at physiological pH, may play an important role in the ability to form hydrogen bonds [187-189].

The 26 first aminoacids of two such proteins have been coded, with MW 25.6 and 28 kiloDaltons and correspond to HMG-1 and HMG-2, respectively [194].

### 4. ELEMENTS OF PHARMOKOKINETICS OF cis-DDP PARAPLATIN AND IPROPLATIN.

Cis-DDP and carboplatin are administered intravenously in maximum doses of 100 and 400 mg/m2 respectively, every 4 weeks. The first is administered in a freshly prepared solution in distilled water with mannitol in 1:1 stoichiometry. Paraplatin is diluted with a 5% dextrose solution in water or with a 0.9% NaCl or with distilled water. After 3 hrs from the administration of cis-DDP, about 90% of the drug is bound with the plasma proteins, while in 1 hr, 50% of it is bound [195]. Paraplatin on the other hand is bound 50% only after about 6 hrs (Table I).

**Table I. Comparison of pharmakokinetics of cis-DDP and Paraplatin**

| Half Life | Paraplatin | Cis-DDP |
|---|---|---|
| Free Pt, non bound to proteins | 6 hrs | 1hr |
| Precursor compound | 1.5 hrs | 0.3-0.5 hrs |
| Urine Excretion | | |
| 24 hrs | 70% | 30% |
| 3 days | 80% | -- |
| 5-6 days | -- | 40-50% |

In conclusion, in the case of cis-DDP a prohydration of the patient is needed and the intravenous administration is slow (solubility : 1 mg/mol in 0.9% NaCl solution), requiring often admission of the patient in hospital and consequently, increasing the cost. Toxicity, nausea and vomiting tendency appear and are treated with the administration of high doses of antiemetic agents. On the contrary, with Paraplatin there is no need of prohydration. Owed to higher solubility, the administration is fast and the patient is not staying in hospital. Most important, its toxicity is smaller, with myelosuppression the dose limiting toxicity, while nausea and vomiting are treated with lower doses of antiemetics.

Therefore paraplatin or carboplatin shows comparative advantages than cis-DDP having lower toxicity, thus allowing increase in dose up to 400 mgr/m2 and lower cost of therapy. Also, its aqueous solutions (0.9% NaCl)

are stable for a long time and they should not be freshly prepared before use, like <u>cis</u>-DDP.

The mechanisms, of action of <u>cis</u>-DDP and carboplatin are similar, i.e., carboplatin like <u>cis</u>-DDP react with DNA and form InterSCL and IntraSCL, as well as IntraSCL of DNA-Pt-protein type [196]. In aqueous solutions, the 1,1-cyclobutane dicarboxylic anion is liberated in two steps, with breaking first of one Pt-O bonding and simoultaneous nucleophilic addition of water (or of other ligands, in vivo) with $k_1$=8.0X10$^{-4}$ sec$^{-1}$ at 25$^{o}$C, followed by its complete release with $k_2$=k[H$^+$]$^1$ and k=1.61X10$^{-4}$ dm$^3$mol$^{-1}$s$^{-1}$ (acid catalyzed). The inverse reactions (formation of the chelate) is not taking place in the presence of acid [197].

The rate constant for the overall hydrolysis of carboplatin was estimated to be 7.2X10$^{-7}$s$^{-1}$ in phosphate buffer solution of pH=7 at 37$^{o}$C. As exposed, it seems that the reaction of carboplatin with DNA proceeds in two steps, i.e., formation of a monodentate complex first and a bidentate afterwards. Based on the hydrolysis constants, the quantity of carboplatin required to achieve the same percentage of binding with DNA, as <u>cis</u>-DDP, was calculated to be about 100 times larger than the latter (under similar conditions, k=8X10$^{-5}$s$^{-1}$), although in vivo a 20-40 fold excess seems to be enough [198].

In summary carboplatin, (<u>i</u>) is about 45 times less toxic than <u>cis</u>-DDP, (<u>ii</u>) the IntraSCL that it forms with DNA appear 12 hours later than those of <u>cis</u>-DDP and (<u>iii</u>) the IntraSCL DNA-proteins that it forms show their maximum quantity 6 hours later than the corresponding ones with <u>cis</u>-DDP [199].

$^1$H NMR studies show that cyclobutane is rotating fast around the carboxylate groups, bonded to Pt(II), in carboplatin [200]. It was also shown [201] that carboplatin reacts faster with 5'-GMP (k=4.1X10$^{-6}$s$^{-1}$) than the phosphates (k=4.3X10$^{-7}$s$^{-1}$) and chloride (k=1.2X10$^{-6}$s$^{-1}$) or water (k<5.9 X 10$^{-9}$s$^{-1}$). Once the 1:1 complex of the metal ligand is formed, the formation of the 1:2 complex through N7 is fast (k=1.3X10$^{-5}$s$^{-1}$).

Iproplatin (CHIP) on the other hand containing Pt(IV), acts as a prodrug, to produce the corresponding complex with Pt(II), which then reacts with DNA. This is seen from the fact that for its reaction with DNA in vitro, the addition of a reducing agent like Fe(ClO$_4$)$_2$.6H$_2$O or ascorbic acid is required [202]. The rate constant of the reaction of iproplatin with ascorbic acid is k=0.584 M$^{-1}$s$^{-1}$ at 37$^{o}$C ($t_{1/2}$=1.5 hrs) [203]. For its reaction with 5'-GMP at 37$^{o}$C and 1:1 stoichiometry, k<10dm$^3$mol$^{-1}$h$^{-1}$ (quite slow) and $t_{1/2}$>10$^4$ hrs and consequently its action requires prior reduction to Pt(II). The reaction of its Pt(II) analog with GMP however, have $k_1$=1.69 at 25$^{o}$C and $k_1$=2.96 dm$^3$mol$^{-1}$s$^{-1}$ at 37$^{o}$C (for <u>cis</u>-DDP 1.44 and 2.82 dm$^3$mol$^{-1}$s$^{-1}$ respectively) for the 1:1 complex. For the 1:2 complex, $k'_1$=0.162 at 25$^{o}$C and $k'_1$=0.848 dm$^3$mol$^{-1}$s$^{-1}$ at 37$^{o}$C (for <u>cis</u>-DDP, 0.238 and 0.650 dm$^3$mol$^{-1}$s$^{-1}$) [204, 205], i.e., both are faster reactions.

### 5. CONCLUDING REMARKS.

Despite the great deal of work performed on the subject, there is no definitive conclusion concerning the mechanism of action of cis-DDP, since there are still unanswered questions e.g., the non recognition of the 1,2-IntraSCL by cell repairing mechanisms, while they are related with DNA replication. It can however be argued that the 1,2-IntraSCL may consitute the main damage caused by cis-DDP to DNA, thereby leading to antitumor action.

The ability of cis-DDP to form 1,2-IntraSCL with d(ApG) and d(GpG) and the inability of trans-DDP to act similarly and form only 1,3-IntraSCL with d(GpXpG) as well as InterSCL, may also be the main difference of the two isomers, leading to antitumor action for the former and to toxicity only for the latter. Cis-DDP causes a greater distortion to the secondary structure of DNA, while trans-DDP causes a greater local distortion, i.e., near the platination site.

The conformation of the sugar at 5' may also play an important role in the mutagenic and toxic dose of the drug during the formation of the 1,2-IntraSCL.

### REFERENCES

1. A. Werner, Z. Anorg. Allg. Chemie, 3, 267 (1893).
2. G.H.W. Milburn and M.R. Trutter, J.Chem.Soc.(A), 1608(1966)
3. R.G. Pearson, J. Chem. Educ., 45, 581 (1968)
4. J. Chatt, L.A. Duncanson and L.M. Venanzi, J. Chem. Soc., 4456 (1955)
5. I.I. Chernyayev, Ann.Inst. Platine SSSR, 4, 261 (1926)
6.a. D. Banerjea, F. Basolo and R.G. Pearson, J.Am. Chem. Soc., 79, 4055 (1957)
b. F. Basolo and R.G. Pearson, Progr. Inorg. Chem., 4, 381 (1962)
7. S.S. Zumdahl and R.S. Drago, J.Am.Chem.Soc., 90, 6669 (1968)
8. S.J.S. Kerrison and P.J. Sadler, J. Chem. Soc. Dalton Trans., 2363 (1982)
9. B. Rosenberg, L. van Camp and T.Krigas, Nature, 205, 698 (1965)
10. B. Rosenberg, E. Reinshaw, L.van Camp, J. Hartwick and J. Drobnik, J. Bacteriol, 93, 716 (1967)
11. B. Rosenberg, L. Van Camp, E.B. Gringley and A.J. Thomson, J. Biol. Chem, 242, 1347 (1967)
12. B. Rosenberg, L. van Camp, J.E. Trosko and V.H. Mansour, Nature, 222, 385 (1969)
13. B. Rosenberg, Die Naturwiss, 60, 399 (1973)
14. B. Rosenberg, Biochimie, 60, 859 (1978)
15. See for example, "6th International Symposium on Platinum and Other Metal Coordination Compounds in Cancer Chemotherapy", eds UCSD, San Diego, California, USA, Jan 23-26, 1991, pp. 118,119,126,138,144,146,148 and references sited there in.
16. M.W. Fiorentino and G. Ghiotto, Inorg. Chim. Acta, 137, 59 (1987)
17. M. Green, M. Garner and D.M. Orton, Trans. Met. Chem, 17, 164 (1992)
18. B. Rosenberg, Cancer (Phil.), 55, 2303 (1985)

19. M.J. Cleare and J.D. Hoeschele, Bioinorg. Chem., 2, 187 (1973)
20. T.A. Connors, M. Jones, W.C.J. Ross, P.D. Braddock, A.R. Khokhar and M.L. Tobe, Chem.- Biol. Interactions, 5, 415 (1972)
21. K.P. Beaumont, C.A. Mc Aulliffe and M.J. Cleares, Chem. - Biol. Interactions, 14, 179 (1976)
22. M.J. Cleare, P.C. Hydes, B.W. Malebi and P.M. Watkins, Biochimie, 60, 835 (1978)
23. J.J. Roberts and A.J. Thomson, Progr. Nucl. Acids Res., 22, 71 (1979)
24. M.J. Cleare, P.C. Hydes, D.R. Hepborn and B.W. Malerbi, in "CISPLATIN: Current Status and New Developments", A.W. Prestayko, S.T. Crooke and S.K. Carter eds, N.Y. Academic Press, pages 149-170 (1980).
25. A.J. Thomson, R.J.P. Williams and S. Reslova, Struct. and Bonding, 11, 1 (1972)
26.a. J. Reedijk, A.M.J. Fichtinger - Shepman, A.J. van Osteroom and P. van de Putte, Struct. and Bonding, 67, 53 (1987)
b. A.I. Stetsenko, M.A. Presnov and A.L. Konovalova, Russ. Chem. Rev., 50, 353 (1983)
27. J. Reedijk, Inorg. Chim. Acta, 198, 873 (1992)
28. P. Köpf-Maier and H. Köpf, Die Naturwiss., 73, 239 (1986)
29. N. Farell, S.G. de Almeida and K.A. Skov, J.Am. Chem. Soc., 110, 5018 (1988)
30. N. Farell and Y.Qu, Inorg. Chem, 28, 3416 (1989)
31. N. Farell, Y.Qu and M.P. Hacker, J. Med. Chem., 33, 2179 (1990)
32. A.Peritz, S.Al.Baker, J.F. Vollano, J.E. Schurig, W.T. Brudner and J.C. Dabrowiak, J. Med. Chem., 33, 2184 (1990)
33. N. Farell, D.M. Killey, W.Schmidt and M.P. Hacker, Inorg. Chem., 29, 397 (1990)
34. N. Farell, T.T.B. Ho, J.P. Souchard, F.L. Wimmer, S.Cros and N.P. Johnson, J. Med. Chem., 32, 2240 (1989)
35. N. Farell and M.V. van Beusichem, Inorg. Chem., 31, 634 (1992)
36. N. Farell, L.R. Kelland and M.V. Beusichem, Cancer Res., 52, 5065 (1992)
37. L.S. Hollis, A. R. Amundsen and E.W. Stern, J. Med. Chem., 32, 128 (1989)
38. L.S. Hollis, W.J. Sundquist, J.W. Burstyn, W.J. Heiger - Bernays, S.F. Bella, K.J. Ahmed, A.R. Amundsen, G.W. Stern and S.J. Lippard, Cancer Res, 51, 1866 (1991)
39. L.K. Webster, E.B. Deacon, D.P. Buxton, B.L. Hilcoat, A.M. James, J.A.G. Roos, R.J. Thomson, L.P.G. Wakelin and T.L. Williams, J. Med. Chem., 35, 3349 (1992)
40. P. Caputo, F.P. Intini, G. Natile, M. Coluccia, F. Loseto and A. Nassi in " Greek - Italian Meeting on Chemistry of Biological Systems and Molecular Chemical Enginneering ", Cetraro, Italy, October 6-9, 1992, Proceedings, pages 11-12.
41. M. Colucia, A. Nassi, F. Loseto, A. Boccarelli, M.A. Mariggio, D. Giordano, F.P. Intini, P. Caputo and G. Natile, J. Med. Chem., 36, 510 (1993)
42. N. Farell, " Transition Metal Complexes as Drugs and Chemotherapeutic Agents ", 1st Eds, London, kluwer Publ., 1989, chapt. 2.4.1.
43. P. Horace and J. Drobnik, Biochim. Biophys. Acta, 254, 341 (1971)
44. N. Hadjiliadis and T. Theophanides, Can. J. Spectrosc., 16, 135 (1971)
45. N. Hadjiliadis, P. Kourounakis and T. Theophanides, Inorg. Chim. Acta, 7, 226 (1973)

46. A. Terzis, N. Hadjiliadis, R. Rivest and T. Theophanides, Inorg. Chim. Acta, 12, $L_5$ (1975)

47. N. Hadjiliadis, and T. Theophanides, Inorg. Chim. Acta, 16, 67 (1976)

48. N. Hadjiliadis, and T. Theophanides, Inorg. Chim. Acta, 16, 77 (1976)

49. L. G. Marzilli, Progr. Inorg. Chem., 23 255 (1976)

50. D. J. Hodgson, Progr. Inorg. Chem., 23, 211 (1976)

51. R. B. Martin, " Frontiers in Bioinorganic Chemistry ", eds. Antonio Xavier, Verlag eds., Weinheim, Germany, (1986), pages 71-79.

52. N. Hadjiliadis, Chim. Chron. (Gen. Eds.), 52, 148 (1987)

53. R.B. Martin, Acc. Chem. Res., 18, 32 (1985)

54. B. Lippert, Gazz. Chim. Ital. 118, 153 (1988)

55. B. Lippert, Comments Inorg. Chem., 37, 1 (1989)

56. S.E. Miller, K.J. Gerard and D.A. House, Inorg. Chim. Acta, 190, 135 (1991)

57. D.S. Martin and R.J. Adams in "Advances in the Chemistry of the Coordination Compounds", Macmillan eds, N.Y., 1961, pages 579-589.

58. M.C. Lim and R.B. Martin, J. Inorg. Nucl. Chem., 38, 1911 (1976)

59. D.M. Orton, V. Gretton and M. Green, Inorg. Chim. Acta, 204, 265 (1993)

60. T.G. Appleton, A.J. Bailey, K.J. Barnham and J.R. Hall, Inorg, Chem., 31, 3077 (1992)

61. C.J. Abraham, K.J. Gerard and D.A. House, Inorg Chim Acta, 209, 149 (1993)

62. C.J. Boreham, J.A. Broomhead and O.P. Fairlie, Aust. J. Chem, 34, 659 (1981)

63. T.G. Appleton, R.D. Berry, C.A. Davis, J.R. Hall and H.A. Kimlin, Inorg Chem., 23, 3514 (1984)

64. R. Faggiani, B. Lippert, C.J.L. Lock and B. Rosenberg, Inorg. Chem., 17, 1941 (1978)

65. R.Faggiani, H.E. Howard - Lock, C.J.L. Lock, B. Lippert and B. Rosenberg, Can. J. Chem., 60, 529 (1982)

66. R. Kuroda, S. Neidle, I.M. Ismail and P.J. Sadler, Inorg. Chem., 22, 3620 (1983)

67. P.P. Gately and S.B. Howell, Br. J. Cancer, 67, 1171 (1993)

68. H. Timmer - Bosscha, N.H. Mulder and E.G.E. de Vries, Br. J. Cancer, 66, 227 (1992)

69. S.J. Berners - Price and P.W. Kuchel, J. Inorg Biochem., 38, 305 (1990)

70. S.J. Berners - Price and P.W. Kuchel, J. Inorg Biochem., 38, 327 (1990)

71. R.E. Norman, J.D.Ranford and P.J. Sadler, Inorg. Chem, 31, 877 (1992)

72. R.E. Norman and P.J. Sadler, Inorg. Chem., 27, 3583 (1988)

73. J.D. Bell, R.E. Norman and P.J. Sadler, J. Inorg. Biochem., 31, 241 (1987)

74. R. Wing, H. Drew, T. Takano, C. Broka, S. Takano, O. Ishira and R. Dickerson, Nature 287, 755 (1980)

75. D.E. Thurston and A.S. Thomson, Chem. Brit, 26, 767 (1990)

76. U. Pindur, M. Haber, and K. Sattler, J. Chem. Educ., 70, 263 (1993)

77. O. Kenn and W. Hunder, Angew. Chem. (Int. Ed. Engl) 30, 1254 (1991)

78. S. J. Lippard, Acc. Chem. Res., 11, 211 (1978)

79. N.I. Sundquist and S.J. Lippard, Coord. Chem. Revs., 293 (1990)

80. E.C. Long and J.K. Barton, Acc. Chem. Res., 23, 273 (1990)

81. J.K. Barton, Comments Inorg. Chem., 3, 321 (1985)

82. A.H.J. Wang, J. Nathans, G. van der Marel, J.H. van Boom and A. Rich, Nature, 276, 171 (1978)

83. N. M. Kostic, Comments Inorg. Chem., 8, 137 (1988)

84. H. Masuda and O. Yamauchi, Inorg. Chim. Acta, 136, $L_{23}$ (1987)

85. A. Odani, R. Shimada, H. Masuda and O. Yamauchi, Inorg. Chem., 30, 2133 (1991)

86. O. Yamauchi, A. Odani, R. Shimada and Y. Kosato, Inorg. Chem., 25, 3337 (1986)

87. M.V. Keck and S.J. Lippard, J. Am. Chem. Soc., 114, 3386 (1992)

88. E.C.M. Lempers, J.S. Boshkin and N.M. Kostic, Nucl. Acid. Res., 21, 1983 (1993)

89. A.M. Bray, D.P. Kelly, P.O.L. Moch, R.F. Martin and L.P.G. Wakelin, Inorg. Chem., 43, 629 (1990)

90. R.V. Gessner, G.J. Quigley, A.H.J. Way, G.A. van der Marel, J.H. van Boom and A. Rich, Biochemistry, 24, 237 (1985)

91. M.D. Reily, T.W. Hambley and L.G. Marzilli, J. Am. Chem. Soc., 110, 2999 (1988)

92. M. Krauss, H. Bosch and K.J. Miller, J. Am. Chem. Soc., 110, 4517 (1988)

93. L.G. Marzilli and T. Kistenmaher, Acc. Chem. Res., 10, 146 (1977)

94. H. Sigel, Chem. Soc. Revs., 22, 213 (1993)

95. S.E. Sherman and S.J. Lippard, Chem. Revs., 87, 1153 (1987)

96. J.A. Howle and G.R. Gale, Biochem. Pharmacol, 19, 2757 (1970)

97. G.L. Cohen, W.R. Bauer, J.K. Barton and S.J. Lippard, Science, 203, 1014 (1979)

98. B. Lippert, Biometalls, 5, 195 (1992)

99. A.M.J. Fichtinger - Schepman, J.L. van der Veer, J.H.J. den Hartog, P.H.M. Lohman and J. Reedijk, Biochemistry, 24, 707 (1985)

100. L.A. Zwelling, in "Platinum and Other Metal Chemotherapeutic Agents", ACS Symposium Series, v. 209, Ed. S.J. Lippard, A.C.S., Washington D.C., (1983), pages 27-49

101. S.L. Bruhn, J.H. Toney and S.J. Lippard, Progr. Inorg. Chem., 38, 477 (1990)

102. K. Hemminki and W.C. Thilly, Mutat. Res. 202, 133 (1988)

103. V. Narasimham and A.M. Bryan, FEBS Lett, 54, 49 (1975)

104. P.J. Stone, A.D. Kelman and F.M. Sinex, Nature, 251, 736 (1974)

105. A.D. Kelman and W. Buchbinder, Biochimie, 60, 901 (1978)

106. J.D. Tullins, H.M. Ushay, C-M. Menkel, J.P. Caradonna and S.J. Lippard, in "Platinum and Other Metal Chemotherapeutic Agents", Ed. S.J. Lippard, A.C.S., Washington D.C., (1983), pages 51-74 and references sited therein.

107. J.P. Caradonna and S.J. Lippard, in "Platinum Coordination Complexes in Cancer Chemotherapy", ed. M.P. Hacker, E.B. Douple and I.H. Krakoff, Martinus Nijhoff Publ., Boston, (1984), pages 14-26 and references sited therein.

108. R. Royer - Pukora, L.K. Gordan and W.A. Haseltine, Nucleic Acids Res., 9, 4595 (1981)

109. K. Inagaki, K. Kasuyo and Y. Kidani, Inorg. Chim. Acta, 91, $L_{13}$ (1984)

110. A. Eastman, Biochemistry, 21, 6732 (1982)

111. A. Eastman, Biochemistry, 22, 3927 (1983)

112. A. Forsti, R. Laatikainen and K. Hemminki, Chem. - Biol. Interactions, 63, 1 (1987)

113. J.P. Caradonna, S.J. Lippard, M.J. Gait and M. Singh, J. Am. Chem. Soc., 104, 5793 (1982)

114. J.P. Caradonna and S.J. Lippard, Inorg. Chem., 27, 1454 (1988)

115. C.A. Lepre, L.G.Strethkamp and S.J. Lippard, Biochemistry, 26, 5651 (1987)

116. A. Eastman, M.M. Jemmerwein and D.L. Nagel, Chem. - Biol. Interactions, 67, 71 (1988)

117. A.L. Pinto and S.J. Lippard, Proc. Natl. Acad. Sci. (USA), 82, 4616 (1985)

118. D.P. Bancroft, C.A. Lepre and S.J. Lippard, J.Am Chem. Soc., 112, 6860 (1990)

119. J.H.J. den Hartog, C. Altona, J.H. van Boom, G.A. van der Marel, G.A.G. Haasnoot and J. Reedijk, J. Am. Chem. Soc., 106, 1528 (1984)

120. S.E. Sherman, D. Gibson, A.H.J. Wang and S.J. Lippara, Science, 230, 412 (1985)

121. S.E. Sherman, D. Gibson, A.H.J. Wang and S.J. Lippard, J. Am. Chem. Soc., 110, 7368 (1988)

122. J.H.J. den Hartog, G. Altona, J.C. Chottard, J.- P. Girault, J.-Y. Lallemand, F.A.A.M. de Leeuw, A.T.M. Marcelis and J.Reedijk, Nucl. Acids Res., 10, 4715 (1982)

123. G. Admiraal, J.L. van der Veer, R.A.G. de Graaf, J.H.J. den Hartog and J. Reedijk, J. Am. Chem. Soc., 109, 592 (1987)

124. J.C. Dewan, J. Am. Chem. Soc., 106, 7239 (1984)

125. A. Jack, J.E. Ladner, D. Rhodes, R.S. Brown and A. Klug, J. Mol. Biol, 111, 315 (1977)

126. J. Kozelka, G.A. Petsko, G.J. Quigley and S.J. Lippard, Inorg Chem., 25, 1075 (1986)

127. J. Kozelka, G.A. Petsko, G.J. Quigley and S.J. Lippard, J. Am. Chem., Soc., 107, 4079 (1985)

128. A. Laoui, J. Kozelka and J.-C. Chottard, Inorg, Chem., 27, 2751 (1988)

129. J.A. Rice, D.M. Crothers, A.L. Pinto and S.J. Lippard, Proc. Natl. Acad. Sci. (USA), 85, 4158 (1988)

130. J. Kozelka, S. Archer, G.A. Petsko, S.J. Lippard and G.J. Quigley, Biopolymers, 26, 1245 (1987)

131. F. Herman, J. Kozelka, V. Stoven, E. Guillet, J.P. Girault, T. Huynth - Dinh, J. Igolen, J.-Y. Lallemand and J.-C. Chottard, Eur.J.Biochem. 194, 119 (1990)

132. L.J.N. Bradley, K.J. Yarema, S.J. Lippard and J.M. Essigman, Biochemistry, 32, 982 (1993)

133. D. Burnouf, C. Gouthier, J.-C. Chottard and R.P.D. Fuchs, Proc. Natl. Acad. Sci. (USA), 87, 6087 (1990)

134. A. Forsti, P. Vodicke and K. Hemminki, Chem. - Biol. Interactions, 74, 253 (1990)

135. A. Eastman, Biochemistry, 25, 3912 (1986)

136. J.J. Roberts and F. Friedlos, Chem. - Biol. Interactions, 39, 181 (1982)

137. V.A. Bohr, E. Reed and W. Zhen, in "Platinum and Other Metal Coordination Compounds in Cancer Chemotherapy", ed. S.B. Howell, Plenum Press eds., N.Y., (1991), pages 231-240

138. J.P. Macquet and T. Theophanides, Bioinorg. Chem., 5, 59 (1975)

139. A. Eastman, Biochemistry, 24, 5027 (1985)

140. M. Sip, A. Schwartz, F. Vavelle, M. Ptal and M. Leng, Biochemistry, 31, 2508 (1992)

141. J.M. Pascoe and J.J. Roberts, Biochem. Pharmacol., 23, 1345 (1974)

142. J.M. Pascoe and J.J. Roberts, Biochem. Pharmacol., 23, 1359 (1974)

143. L.C. Erickson, L.A. Zwelling, J.M. Ducore, W.A. Sharley and W. Kohn, Cancer Res., 41, 2791 (1981)

144. L.A. Zwelling, T. Anderson, and W. Kohn, Cancer Res., 39, 365 (1979)

145. P. Gibson and S.J. Lippard, Inorg. Chem, 26, 2275 (1987)

146. J.H.J. den Hartog, C. Altona, J.H. van Boom, A.T.M. Marcelis, G.A. van der Marel, L.J. Rinkle, G. Wille - Hazeleger and J. Reedijk, Eur. J. Bioclem, 134, 485 (1983)

147. C.A. Lepre, L. Chassot, C.E. Castello and S.J. Lippard, Biochemistry, 29, 811 (1990)

148. H.M. Ushay, T.D. Tullius and S.J. Lippard, Biochemistry, 20, 3744 (1981)

149. K.M. Comess, C.E. Castello and S.J. Lippard, Biochemistry, 29, 2102 (1990)

150. J.P. Maquet and T. Theophanides, Inorg. Chim Acta, 18, 185 (1976)

151. G. Pneumatikakis, N. Hadjiliadis and T. Theophanides, Inorg. Chim Acta, 22, $L_1$ (1987)

152. G. Pneumatikakis, N. Hadjiliadis and T. Theophanides, Inorg. Chem., 17, 915 (1978)

153. N. Hadjiliadis and G. Pneumatikakis, J. Chem Soc. Dalton Trans., 1691 (1978)

154. D. Cozak, A. Mardley, M.J. Oliver and A.L. Beauchamp, Inorg Chem., 25, 2600 (1986)

155. A.L. Beauchamp and F. Belanger-Gariepy, Inorg. Chim. Acta, 124, $L_{23}$ (1986)

156. E. Colacio, J. Suarez - Varela, J.M. Dominguez - Vera, J.C. Avila-Roson, M.A. Hidalgo and D. Martin-Ramos, Inorg. Chim. Acta, 202, 219 (1992)

157. J. Lorberth, W. Massa, M.E. Essawi and L. Labib, Angew, Chem. (Int. Ed. Engl.), 27, 1160 (1988)

158. G. Frommer, H. Preut and B. Lippert, Inorg. Chim. Acta, 193, 111 (1992)

159. B. Lippert, H. Schollhorn and U. Thewalt, J. Am. Chem. Soc., 105, 6616, (1986)

160. C.S. Fonts, M.D. Reily, L.G. Marzilli and G. Zon, Inorg. Chim. Acta, 137, 1 (1987)

161. L.G. Marzilli, C.S. Fonts, T.P. Kline and G. Zon, in "Platinum and Other Metal Coordination Compounds in Cancer Chemotherapy", Ed. M. Nicolini and G. Bandoli, It. Chem. Soc, Albano Terme (Padua), Italy, 1987, pp. 75

162. P.G. Yohannes, G. Zon, P.W. Doetsch and L.G. Marzilli, J. Am. Chem. Soc., 115, 5105 (1993)
163. M. Green and J.M. Miller, J. Chem. Soc. Chem. Commun., 1864 (1987)
164. A. Nikolaou, V. Theodorou and N. Hadjiliadis, Inorg. Chim. Acta, 208, 91 (1993)
165. A. Arrick and C.F. Nathan, Cancer Res, 44, 4224 (1984)
166. A. Eastman, Chem. - Biol. Interactions, 61, 241 (1987)
167. B.J. Carter, Inorg. Chim. Acta, 137, 125 (1987)
168. B. Odenheimer and W. Wolf, Inorg. Chim. Acta, 66, L41 (1982)
169. J. Filipski, K.W. Kohn and W.M. Bonner, FEBS Letters 152, 105 (1983)
170. J.J. Huyes and W.M. Scovell, Biochim. et Biophys. Acta, 1088, 413 (1991)
171. L.A. Zwelling, S. Michaels, H. Schwartz, P.P. Dobson and K.W. Kohn, Cancer Res, 41, 640 (1981)
172. F. Olinski, A. Nedrychowski, W.N. Schmidt, R.C. Briggs and L.S. Hnilica, Cancer Res, 47,201 (1987)
173. G.A. Petsko, Methods Enzymol., 114, 147 (1985)
174. G.A. Persko, D.C. Philips, R.J.P. Williams and I.A. Wilson J. Mol. Biol., 120, 345 (1978)
175. W.M. Scovell, N. Murhead and L.R. Kroos, Biochem. Biophys. Res. Commun., 142, 826 (1987)
176. Z.M. Banjar, L.S. Hnilica, R.C. Briggs, J. Stein and G. Stein, Biochemistry, 23, 1921 (1984)
177. R.B. Ciccarelli, M.J. Solomon, A. Varsavsky and S.J. Lippard, Biochemistry, 24, 7533 (1985)
178. L.A. Zwelling, J. Filipski and K.W. Kohn, Cancer Res., 39, 4989 (1979)
179. L.A. Zwelling, T. Anderson and K.W. Kohn, Cancer Res., 39, 356 (1979)
180. ACM Ploy, A. van Dijk and P.H.M. Lohmann, Cancer Res., 44, 2043 (1984)
181. A.J. Fornace and P.S. Seres, Mutation Res., 94, 277 (1982)
182. A. Eastman and M.A. Barry, Biochemistry, 26, 3303 (1987)
183. M.A. Tukalo, M-D Kubler, D. Kern, M. Mongel, C. Ehresmann, J.-P. Ebel, B. Ehresmann and R. Giege, Biochemistry, 26, 5200 (1987)
184. T. Le Doan, M. Guigues, J.-J Toulme and C. Helene, Biochim. Biophys. Acta, 825, 353 (1985)
185. B.A. Donahue, M. Augot, S.F. Bellon, D.K. Treiber, J.H. Toney, S.J. Lippard and J.M. Essigmann, Biochemistry, 29, 5872 (1990)
186. S.F. Bellon, J.H. Coleman and S.J. Lippard, Biochemistry, 30, 8026 (1991)
187. M.E. Bianchi, M. Beltrane and G. Paonesca, Science, 243, 1056 (1989)
188. D.M.J. Lilley, Nature, 357, 282 (1992)
189. R. Baum, Chem. Engineer. News, 19 (1992)
190. B.L. Vallee and P.S. Auld, Acc. Chem. Res., 26, 543 (1993)
191. J.M. Berg, Inorg Chem, 37, 144 (1989)
192. B.D. Rhodes and R. Klug, in "Spectrum der Wissenschaft", p. 54 (1993)
193. N.P. Pavlevitch and C.O. Pabo, Nature 253, 809, (1991)
194. P.E. Nichen, C. Jeppeson and O. Bucharest, FEBS Lett, 235, 122 (1988)
195. A.W. Prestayko, in "CISPLATIN: Current Status and New Developments", Eds A.W. Prestayko, S.T. Crooke and J.K. Carter, N.Y. Academic Press, 1980, pages 1-7
196. R.J. Knox, F. Friedlos, D.A. Lyndall and J.J. Roberts, Cancer Res., 46, 1972 (1986)

197. L. Canovese, L. Cattalini, G. Chessa and M.L. Tobe, J. Chem. Soc. Dalton Trans., 2135 (1988)
198. R.J. Knox, F. Friedlos, D.A. Lyndall and J.J. Roberts, Cancer Res., 46, 1972 (1986)
199. K.C. Micetich, D. Barnes and L.C. Erickson, Cancer Res., 45, 4043 (1985)
200. S. Neidle, I.M. Ismail and P.J. Sadler, J. Inorg. Biochem, 13, 205 (1980)
201. U. Frey, J.D. Ranford and P.J. Sadler, Inorg. Chem., 32, 1333 (1993)
202. E.E. Blatter, J.F. Vollano, B.S. Krishnan and J.C. Dabrowiak, Biochemistry, 23, 4817 (1984)
203. D.J. Evans and M. Green, Inorg. Chim. Acta, 130, 183 (1987)
204. D.J. Evans and M. Green, J. Chem. Soc. Chem. Commun., 124 (1987)
205. R.J. Brandon and J.C. Dabrowiak, J. Med. Chem., 27, 861, (1984)

**Received: February 8, 1995 - Accepted: February 20, 1995 - Received in revised camera-ready format: April 11, 1995**

